# Prolonged expression of the BX1 signature enzyme is associated with a recombination hotspot in the benzoxazinoid gene cluster in *Zea mays*

**DOI:** 10.1093/jxb/erv192

**Published:** 2015-05-11

**Authors:** Linlin Zheng, Michael D. McMullen, Eva Bauer, Chris-Carolin Schön, Alfons Gierl, Monika Frey

**Affiliations:** ^1^Lehrstuhl für Genetik, Wissenschaftszentrum Weihenstephan, Technische Universität München, 85354 Freising, Germany; ^2^USDA ARS, University of Missouri, Columbia, MO 65211, USA; ^3^Lehrstuhl für Pflanzenzüchtung, Wissenschaftszentrum Weihenstephan, Technische Universität München, 85354 Freising, Germany

**Keywords:** Allele-specific expression, biosynthetic cluster, *cis*-element, DIMBOA, defence, QTL mapping, secondary metabolites.

## Abstract

Benzoxazinoids represent preformed protective and allelopathic compounds. The main benzoxazinoid in maize (*Zea mays* L.) is 2,4-dihydroxy-7-methoxy-1,4-benzoxazin-3-one (DIMBOA). DIMBOA confers resistance to herbivores and microbes. Protective concentrations are found predominantly in young plantlets. We made use of the genetic diversity present in the maize nested association mapping (NAM) panel to identify lines with significant benzoxazinoid concentrations at later developmental stages. At 24 d after imbibition (dai), only three lines, including Mo17, showed effective DIMBOA concentrations of 1.5mM or more; B73, by contrast, had low a DIMBOA content. Mapping studies based on Mo17 and B73 were performed to reveal mechanisms that influence the DIMBOA level in 24 dai plants. A major quantitative trait locus mapped to the *Bx* gene cluster located on the short arm of chromosome 4, which encodes the DIMBOA biosynthetic genes. Mo17 was distinguished from all other NAM lines by high transcriptional expression of the *Bx1* gene at later developmental stages. *Bx1* encodes the signature enzyme of the pathway. In Mo17×B73 hybrids at 24 dai, only the Mo17 *Bx1* allele transcript was detected. A 3.9kb *cis*-element, termed DICE (distal *cis*-element), that is located in the *Bx* gene cluster approximately 140kb upstream of *Bx1*, was required for high *Bx1* transcript levels during later developmental stages in Mo17. The DICE region was a hotspot of meiotic recombination. Genetic analysis revealed that high 24 dai DIMBOA concentrations were not strictly dependent on high *Bx1* transcript levels. However, constitutive expression of *Bx1* in transgenics increased DIMBOA levels at 24 dai, corroborating a correlation between DIMBOA content and *Bx1* transcription.

## Highlight

High transcript levels of the signature gene *Bx1* increase concentrations of the defence compound DIMBOA in older maize, and *cis*-elements upstream of *Bx1* are required for high mRNA levels.

## Introduction

Benzoxazinoids, or cyclic hydroxamic acids, have a long record as defence chemicals in poaceous plants. In 1955, benzoxazinoids were described as antifungal compounds in rye seedlings (Virtanen and Hietala, 1955) and in 1959 benzoxazinoids were reported in maize and wheat (Wahlroos and Virtanen, 1959). In the following years, the impact of benzoxazinoids on tolerance against microbial pathogens, herbivores, and as an allelochemical has been documented (Niemeyer, 1988, 2009; Sicker *et al.*, 2000). In maize, control of the European corn borer (*Ostrinia nubilalis*) is of great importance, and the content of 2,4-dihydroxy-7-methoxy-1,4-benzoxazin-3-one (DIMBOA), the main benzoxazinoid in maize, has been successfully increased by breeding programmes (Klun *et al.*, 1970; Grombacher *et al.*, 1989). DIMBOA represents a constitutive defence chemical that is produced without external challenge in the young plant, with concentrations of up to 30mM in the seedling. In addition, benzoxazinoids are specifically induced by herbivory (Dafoe *et al.*, 2013; Huffaker *et al.*, 2013). The constitutive concentration in the plant decreases with age, and high concentrations of DIMBOA (1.5mM, equal to about 1mg g^–1^ of fresh weight; Long *et al.*, 1975, 1977; Campos *et al.*, 1989) are required for biological control. It would be beneficial for plant protection to extend significant DIMBOA levels to later developmental stages of the plant.

**Fig. 1.**
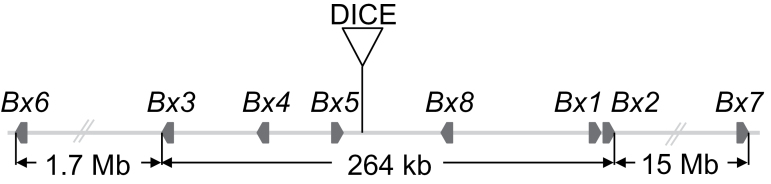
Schematic presentation of the *Bx* gene cluster on chromosome 4. *Bx1* and *Bx2* are separated by 2.5kb. DICE is a 3.9kb *cis*-element influencing transcriptional regulation of *Bx1* and was detected in this study. It is located at position 3 113 226, about 0.5kb downstream of *Bx5.*

The biosynthesis of benzoxazinoids in maize has been elucidated (Frey *et al.*, 1997; von Rad *et al.*, 2001; Frey *et al.*, 2003; Jonczyk *et al.*, 2008; Meihls *et al.*, 2013). The genes *Bx1*–*Bx5* are sufficient to synthesize 2,4-dihydroxy-1,4-benzoxazin-3-one (DIBOA). BX1 is the signature enzyme of the pathway and generates the branch off from the primary metabolism catalysing the formation of free indole that is consecutively hydroxylated by the four specific cytochrome P450 enzymes BX2–BX5. The unstable bioactive benzoxazinoid is stabilized by glucosylation catalysed by the UDP-glucosyl transferase BX8 or BX9. In some grasses, such as wild barley, DIBOA-glucoside is the major benzoxazinoid (Frey *et al.*, 2009; Niemeyer, 2009). The genes *Bx1*–*Bx5* and *Bx8* have been isolated for wheat (Nomura *et al.*, 2003) and wild barley, *Hordeum lechleri* (Grün *et al.*, 2005) and can be considered the core genes of benzoxazinoid biosynthesis. Interestingly, these five genes constitute a biosynthetic cluster and are located within 264kb (corresponding to 6 cM) on chromosome 4 in maize (Fig. 1). The local recombination rate in this region is 22.7 cM Mb^–1^, which is more than 8-fold higher than the average genome-wide recombination rate of 2.7 cM Mb^–1^, assuming a genome size of 2.3 Gb (Schnable *et al.* 2009) and a map length of 6243 cM (IBM302 map from MaizeGDB). In maize, DIBOA-glucoside is further modified by hydroxylation and methylation to yield DIMBOA-glucoside. The respective genes, the 2-oxoglutarate-dependent dioxygenase *Bx6* and the *O*-methyltransferase *Bx7*, locate 1.7Mb upstream and 15Mb downstream of the core cluster. The *Bx8*-homologous gene *Bx9* resides on chromosome 1. All *Bx* genes are highly expressed in seedlings, which have, in parallel, the highest DIMBOA concentrations of any maize growth stage.

With the exception of the UDP-glucosyl transferases, the transcription level of all other *Bx* genes decreases within 3 weeks after imbibition (von Rad *et al.*, 2001).

Gene clustering in plant secondary or specialized metabolism was first demonstrated for *Bx* genes (Frey *et al.*, 1997) and since then has been discovered in several plant species; currently, at least 15 biosynthetic gene clusters have been reported (reviewed by King *et al.*, 2014; Nützmann and Osbourn, 2014). It was speculated that gene clusters might have a selective advantage, since superior allelic combinations are inherited preferentially once established in the coupling phase (Field and Osbourn, 2012; Takos and Rook, 2012). Clustering might also facilitate co-ordinated regulation by domains for chromatin modification. Indeed, for the avenacin biosynthetic cluster, co-ordinated localized chromatin modification was revealed by DNA fluorescence *in situ* hybridization and correlated with expression of clustered genes (Wegel *et al.*, 2009). Data about transcription factors and *cis*-elements that are employed in biosynthetic cluster regulation are scarce. A unique example is given for the momilactone and phytocassene gene cluster in rice (Okada *et al.*, 2009) where transcription is regulated by a chitin oligosaccharide elicitor-inducible basic leucine zipper transcription factor. Nomura *et al.* (2008) identified by biomathematical comparison putative *cis*-elements for *Bx* gene regulation in wheat. However, experimental proof is limited. Regulatory *cis*-elements can be quite distal to the coding region of genes and therefore difficult to determine. Such an arrangement has been detected for the maize *Vgt1*, *tb1*, and *b1* genes, which have essential regulatory sequence elements at a distance of several 10kb. These sequences have been detected by genetic analysis (Stam *et al.*, 2002; Clark *et al.*, 2006; Salvi *et al.*, 2007).

Quantitative trait locus (QTL) analysis has been used to study maize resistance to European corn borer and aphids (Jampatong *et al.*, 2002; Cardinal *et al.*, 2006; Betsiashvili *et al.*, 2015). These studies indirectly addressed the variation in DIMBOA content at later developmental stages, and all detected a major QTL in the region of the *Bx* gene cluster on chromosome 4. In a recent QTL analysis, the DIMBOA content at 32 d after imbibition (dai) in maize plants was assayed directly (Butrón *et al.* 2010) across genetically divergent maize inbred lines. A major QTL was detected in the region of the *Bx* gene cluster with eight recombinant inbred line (RIL) populations of the nested association mapping (NAM) population array (Yu *et al.*, 2008). The parallel association mapping with the *Bx* candidate genes placed a major polymorphism in proximity to the *Bx1* gene.

In this study, we screened all 26 NAM parental lines to evaluate potential differences in DIMBOA content in 24 dai maize plants. QTL mapping was performed using lines with high (Mo17) or low (B73) 24 dai DIMBOA content as parental lines. We specifically characterized the *Bx1*-specific transcription pattern and detected a 3.9kb *cis*-element approximately 140kb upstream of *Bx1* that is required for high *Bx1* transcript levels during the later stages of plant development. A high recombination frequency was associated with the *Bx* gene cluster, suggesting a genetic mechanism that provides diversity in defence gene expression.

## Materials and methods

### Standards, reference chemicals, oligonucleotides

The benzoxazinoids were a gift from Professor D. Sicker, University of Leipzig, Germany, or were prepared as described by von Rad *et al.* (2001). Oligonucleotides were synthesized by Eurofins MWG Operon (Ebersberg, Germany), Microsynth AG (Balgach, Switzerland) and Biomers (Ulm, Germany).

### Bacterial artificial chromosome (BAC) resources

BAC AC213878 (B73) was provided by the Children’s Hospital Oakland Research Institute, CA, USA. BAC b0506A16 (Mo17) was provided by Dr Bailin Li, DuPont Pioneer, USA.

### Plant material

The maize inbred lines B73 and Mo17, the NAM founder lines, the IBM302 population, RILs, and near-isogenic lines (NILs) were provided by the Maize Genetic Stock Center, and Dr Nathan Springer (University of Minnesota). HiII parental lines were provided by Dr Patrick S. Schnable, Iowa State University, IA, USA.

### Molecular biology methods

DNA and RNA isolation, cDNA synthesis, cloning, and PCR amplification was as described by Schullehner *et al.* (2008). Quantitative reverse transcriptase PCR (qRT-PCR) was carried out with a Roche LightCycler 480 instrument using a Bioline SensiFAST™ SYBR No-ROX kit. Primer and PCR conditions are given in Supplementary Table S1, available at *JXB* online. DNA sequencing was by Source (Bioscience, Berlin), GATC-Biotech AG (Konstanz, Germany), and Eurofins Genomics (Ebersberg, Germany). All primer pairs were checked for specificity against the maize B73 reference genome, version 2, and PCR products were sequenced to verify amplification of the respective *Bx* gene.

### Genotyping

Genotypes were determined by the size difference of the PCR products, by using allele-specific primer pairs or by sequencing. Genotyping of the distal *cis*-element (DICE) was by a combination of allele-specific primers and sequencing. The PCR primer pair DICEFW and DICE2REV (Supplementary Table S1) were used for the Mo17 and B73 alleles of DICE and a fragment of about 527bp was amplified independent of the genotype. The fragment included the border of DICE-A and DICE-B and sequence alterations that distinguished the B73 and Mo17 DICE sequences. If duplication was present, the PCR amplicon included two fragments. In this case, the sequencing led to double labelling due to the sequence difference in the two DICE-A parts in Mo17. If a single DICE was present, sequence analysis allowed determination of the DICE-B genotype and discrimination between B73 DICE-A and the second Mo17 DICE-A on the other hand. To discriminate between B73 DICE-A and the first Mo17 DICE-A, the marker M143 was used, which revealed the size differences existing between the two sequences.

### Determination of expression levels by sequencing of PCR-amplified cDNA

Primer pairs Bx1QF2/Bx1QREV2 and Bx2QF/Bx2QR (Supplementary Table S1) were used for the amplification of cDNA and genomic DNA templates. Contamination of cDNA by genomic DNA was recognized by the presence of introns. As controls, *Bx1* was analysed in parallel, and genomic DNA of the B73×Mo17 hybrid was used as a control for both *Bx1* and *Bx2* analyses.

### Maize transformation

Transgenic maize was generated as described by Frame *et al.* (2002). The *Bx1* coding sequence under the control of a maize ubiquitin promoter (Christensen and Quail, 1996) was inserted into Ti-plasmid pTF101.1. pTF101.1 was kindly provided by the Plant Transformation Facility (Iowa State University, Ames, IA, USA). Selfed T1 progeny segregating for wild-type and transgenic plants was analysed. The genotype was confirmed by analysis of *Bx1* transcription (Supplementary Table S1).

### Plant growth and harvesting of plant material

Maize seed was sterilized with 1.3% sodium hypochlorite, washed with tap water and germinated at 28 °C on germination paper for 2–5 d in the dark before analysis or planting (standard soil ED73 with 10%, w/w, sand, 8×8cm pots). Twenty plants were grown in a tray in the plant growth chamber (Climate Chamber HPS 2000; Heraeus Vötsch Industrietechnik GmbH, Germany) for 16h at 22 °C in the light and 8h at 18 °C in the dark at 80% moisture. For each experiment, B73 and Mo17 plants were grown in parallel to RILs, NILs, recombinants, and hybrids, and the plants were placed randomly in the trays. The trays were circulated every 24h.

Shoots of etiolated seedlings were harvested at 4 dai. For the 24 dai material, the blade of the third leaf was cut at the border to the leaf sheath with the scissors. Three to four harvested leaves of one line were pooled, the weight was determined, and the material was frozen immediately in liquid nitrogen and stored at –70 °C.

### Biochemical analysis of benzoxazinoids

Benzoxazinoid extraction was carried out as described by Grün *et al.* (2005). Briefly, the fresh weight of the plant material used was determined (about 400mg of ground powder per assay). The DIMBOA amount was determined by high-performance liquid chromatography (HPLC) using a standard curve. DIMBOA (1 mmol per 1kg of fresh weight) was considered to be 1mM DIMBOA. The ground material was suspended in 3.3 vols of H_2_O (w/v) and incubated for 1h at room temperature to allow the maize β-glucosidase to generate the benzoxazinoid aglucone. No attempt was made to analyse aglucone and glucoside separately. The probes were dissolved in methanol.

HPLC analysis of DIMBOA was carried out on a LiChroCART^®^ 250–4 LiChrospher^®^ 100 RP-18e (5 μm) column (Merck, Darmstadt, Germany) using Dionex 2284 Softron SP2 (Thermo Scientific Dionex). As mobile phases, 0.3% formic acid and methanol were used at a flow rate of 1.0ml min^–1^. The HPLC was run for 60 s in 80% 0.3% formic acid, 20% methanol, and the methanol concentration was then increased to 40% within 300 s and to 42.5% in the following 360 s. Washing of the column was for 60 s with 100% methanol and equilibration was for 180 s with 80% 0.3% formic acid and 20% methanol. Analysis was done with the chromatography data system Chromeleon version 6.80 (Thermo Scientific Dionex). Benzoxazinoid concentration was calculated using a calibration curve. The values were normalized to material fresh weight.

### Statistical analysis tools

Microsoft Excel was used to calculate the average values, standard deviations of the DIMBOA contents, and late *Bx1* expression level. Composite interval QTL mapping (Jansen and Stam, 1994; Zeng, 1994) for the IBM population and selected IBM subpopulation was performed using Windows QTL Cartographer 2.5. Significance thresholds were determined by 1000 permutations (Basten *et al.*, 1997). A high-density genetic map with 1435 markers combining the genotypes of the IBM302 population (Lee *et al.*, 2002) provided by the maize mapping project (http://curation.maizegdb.org/ibm302scores.html) with single-nucleotide polymorphism (SNP) scores from University of Missouri was used (Supplementary material, available at *JXB* online). The total length of this genetic map was 6242.7 cM.

Analysis of variance was performed, and differences between genotypic means were tested with a Kruskal–Wallis test accounting for multiple comparisons at the 0.05 significance level. Allele-specific effects of hybrids were tested using Student’s *t*-test with *P*<0.05 considered significant. Spearman’s correlation coefficient between late DIMBOA content and *Bx1* expression in selected RILs and the two NILs was calculated in R (http://www.r-project.org).

BLAST analysis and genome browsing were performed with the tools of MaizeGDB (http://www.maizegdb.org/, Monaco *et al.*, 2013). All sequence positions are given according to the Arabidopsis Genome Initiative (AGI) B73 RefGen_v2.

## Results

### Effective benzoxazinoid levels beyond the seedling stage are rare in maize

**Fig. 2.**
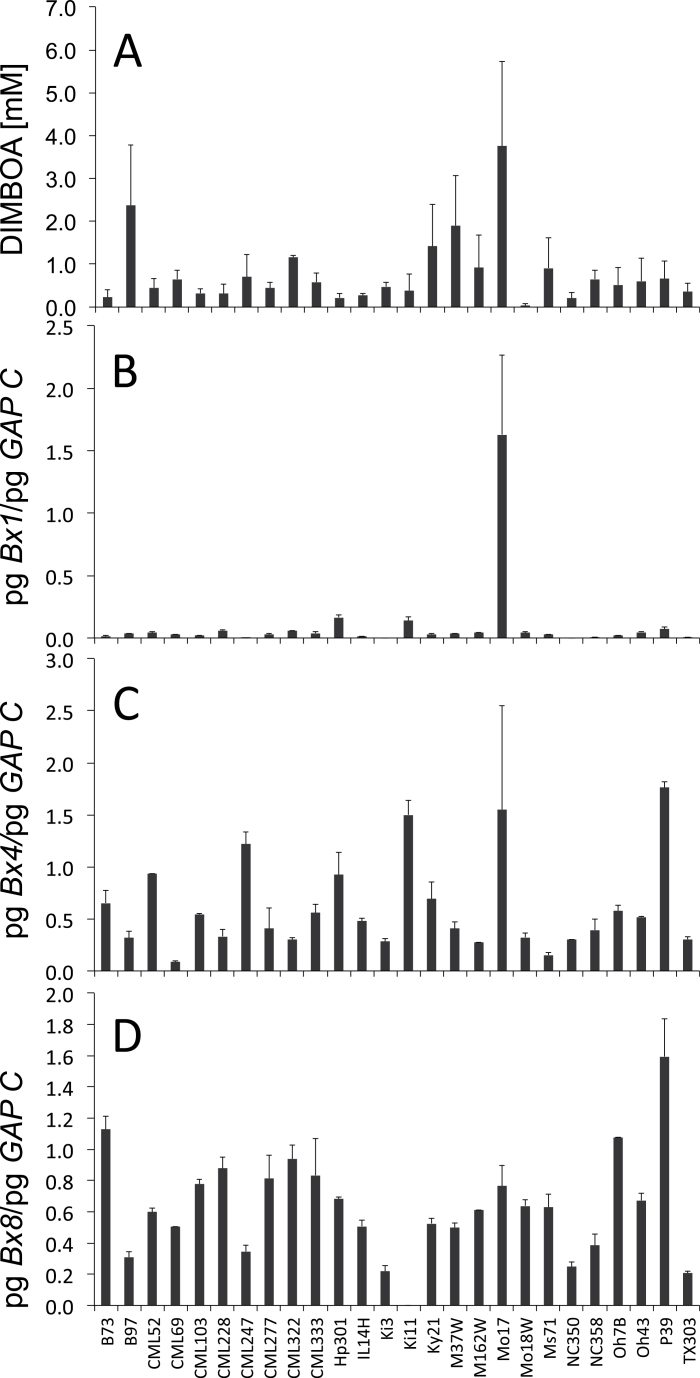
Analysis of 24 dai plants of the NAM founder line panel. (A) DIMBOA content. (B) *Bx1* transcript level. (C) *Bx4* transcript level. (D) *Bx8* transcript level. Transcript levels were normalized to the cytosolic glyceraldehyde-3-phosphate dehydrogenase (*GAPC*) mRNA level. Two sets of material were grown and at least one sample of pooled leaf material (three to four plants; results show means±standard deviation) was analysed.

The first analyses of benzoxazinoid levels in maize (e.g. Long *et al.*, 1975) revealed that, at the seedling stage, concentrations are generally high and significant line differences do not become obvious until the plant reaches heights of about 25cm. We used the NAM panel of maize lines (Yu *et al.*, 2008) to define the range of benzoxazinoid content in older plants that exists due to genetic diversity in maize. To minimize environmental differences, the plants were placed in growth chambers and analysed at 24 dai. At this time point, all lines had a similar size and for all genotypes the third leaf was fully expanded; the blade of this leaf was harvested for benzoxazinoid analysis and RNA extraction. In parallel, 4 dai seedlings of the NAM inbred lines were analysed. At 4 dai, all lines had high benzoxazinoid content in seedling shoots and roots (16.8±9.4 and 7.6±5.7mM, respectively), and the variation within the lines was high (Supplementary Fig. S1, available at *JXB* online). Differences between the lines become obvious for the 24 dai leaf material. For most lines, the concentration was below 1mM (Fig. 2); only lines B97, M37W, and Mo17 reached levels of 2mM or higher. Mo17 had a consistently high benzoxazinoid concentration at 24 dai (3.8±2.0mM; Fig. 2A).

### High late transcript level of the signature gene *Bx1* is a unique feature of Mo17

To obtain a first insight into the transcriptional expression of the biosynthetic genes (*Bx* genes) within the NAM panel, the 24 dai transcript levels of *Bx1* (Fig. 2B), *Bx4* (Fig. 2C), and *Bx8* (Fig. 2D) were determined by qRT-PCR. The three genes represent the signature enzyme (branch-point reaction), a pathway-specific P450 mono-oxygenase, and the UDP-glucosyltransferase required for stabilization of the benzoxazinoid aglucone. Sequence information was available for these genes from all 26 lines of the NAM panel, and primer pairs for all the different alleles were designed. *Bx4* and *Bx8* transcript levels varied between the lines but displayed no correlation with benzoxazinoid levels (Fig. 2C, D; *P*>0.05). *Bx1* mRNA was present at low concentrations in most lines [<0.1 pg pg^–1^ of cytosolic glyceraldehyde 3-phosphate dehydrogenase (GAPC) mRNA] and the level did not correlate significantly with DIMBOA content (*P*>0.5). Mo17 was exceptional in having about a 10-fold higher *Bx1* transcript level compared with all other lines (Fig. 2B, and see below). The data indicated that the mRNA level of the pathway genes is not necessarily correlated with elevated benzoxazinoid concentrations at 24 dai. However, the relatively high *Bx1* transcript level in 24 dai Mo17 could indicate an influence on DIMBOA content in this line.

### *Bx1* transcription is Mo17 allele specific at later developmental stages

**Fig. 3.**
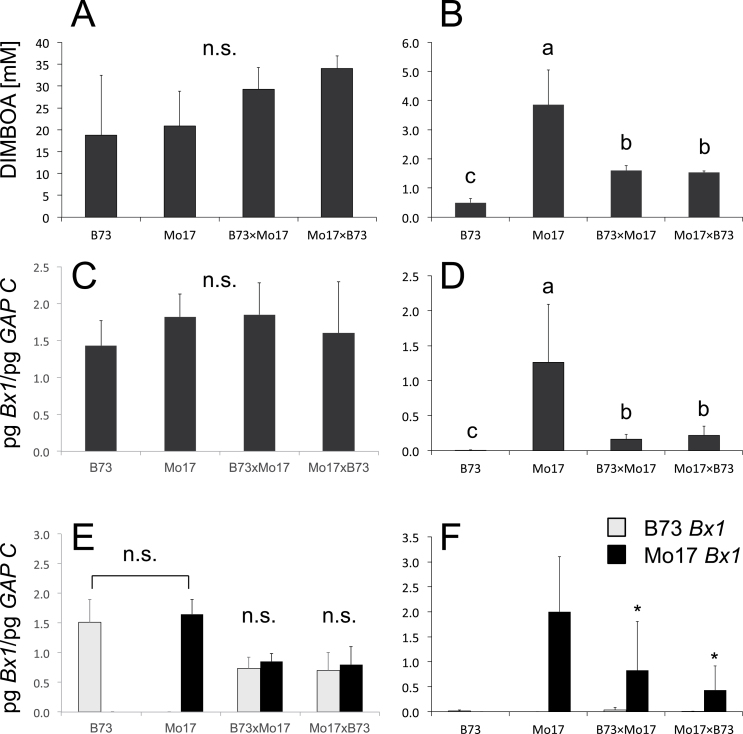
Analysis of B73, Mo17, and hybrids in seedling shoots at 4 dai (A, C, E) and in 24 dai plants (B, D, F). (A, B), DIMBOA content. (C, D) *Bx1* transcript levels. (E, F) Allele-specific analysis of *Bx1* transcript levels; light grey shading indicates the transcript level of the B73 *Bx1* allele and black indicates the transcript level of the Mo17 *Bx1* allele.Transcript levels were normalized to *GAPC.* At least four samples of pooled leaf material (three to four plants) were analysed. Statistical analysis in (A–D) was by Kruskal–Wallis test and multiple comparison of treatments; identical letters above columns indicate no statistical difference (*P*>0.05). In (E) and (F), Student’s *t*-test was used. *P<0.05. Mean values and standard deviation are indicated. n.s., Not significant.

Benzoxazinoid content and transcript levels of the *Bx* genes in Mo17 and B73 as representatives of high and low 24 dai benzoxazinoid-containing lines were analysed in more detail (Fig. 3 and Supplementary Fig. S2, available at *JXB* online). Primer pairs for all alleles of all *Bx* genes in the two lines were designed (Supplementary Table S1). In seedlings (4 dai) of both lines, the pattern for all *Bx* genes was almost identical (Supplementary Fig. S2). In B73, the *Bx1* level decreased to the detection limit by 14 dai. By contrast, *Bx1* levels were high in Mo17 at 24 dai. The high *Bx1* transcription level at this ‘late’ stage was the major difference between the lines in transcript pattern and correlated with the difference in DIMBOA content determined for the two lines at 24 dai (Fig. 3B). SNPs in the coding region of the Mo17 and B73 *Bx1* alleles do not change the amino acid sequence and catalytic properties should be equivalent.

This difference might be caused by the presence of line-specific transcription factors and variation in *cis*-elements. To distinguish between these possibilities, allele-specific primers were designed (Supplementary Table S1), and hybrid plants were analysed (Fig. 3E, F) to determine the contribution of the parental genomes to *Bx1* expression. In 4 dai seedlings, the *Bx1* transcript level was similar in the parental lines and the reciprocal hybrids (Fig. 3C). Allele-specific analysis shows that both alleles were expressed at the same level (Fig. 3E). The picture changed for 24 dai material. In both reciprocal hybrids, the Mo17 allele was expressed almost exclusively (Fig. 3F). Hence, *cis*-element(s) contribute to the 24 dai expression of the Mo17 *Bx1* allele. Since the mid-parent *Bx1* transcript level was not fully reached in the hybrids (Fig. 3D), a negative effect by *trans*-acting factors cannot be excluded.

In summary, 24 dai Mo17 and B73 plants differed in DIMBOA concentration and *Bx1* mRNA levels. The 24 dai expression of the Mo17 allele is probably the result of the interaction of *trans*-factors with Mo17-specific *cis*-element(s). To analyse the impact of transcriptional regulation on benzoxazinoid concentration and to reveal additional mechanisms, a QTL analysis based on the B73×Mo17 intermated RIL population IBM302 (Lee *et al.*, 2002) was performed.

### Mapping reveals a major QTL for prolonged DIMBOA content close to the *Bx* gene cluster

Late DIMBOA concentrations were measured for the IBM302 RILs. As expected for quantitative traits, continuous values of benzoxazinoid content were displayed. The lowest line (MO382) had a benzoxazinoid level close to the detection limit. The value of the highest line (MO147) exceeded the level of the high parent Mo17 by a factor of 4 (Supplementary Fig. S3, available at *JXB* online).

The genotype scores of the IBM302 population provided by the maize mapping project (http://curation.maizegdb.org/ibm302scores.html, Schaeffer *et al.*, 2011) were used to construct a high-density genetic map with 1435 markers (Supplementary material) for QTL mapping. Three QTLs, *QTL1* (chromosome 1), *QTL4-1* (chromosome 4), and *QTL5* (chromosome 5), were detected and were consistent in 1000 permutations (Supplementary Fig. S4, available at *JXB* online). A further QTL on chromosome 4 (*QTL4-2*) had a LOD of 2.7. *QTL4-1* mapped to the short arm of chromosome 4 and overlapped with the *Bx* gene cluster; the major peak was around 200kb upstream of *Bx1* (position 3 109 838). *QTL4-1* (LOD value 15.2) explained 21.5% of the phenotypic variation (Supplementary Table S2, available at *JXB* online). The other three QTLs accounted for 3.3–6.1% of the phenotypic variation (Supplementary Table S2). For *QTL4-1*, *QTL4-2*, and *QTL5*, Mo17 alleles increased the trait value, while at *QTL1* the positive contribution was from B73 (Supplementary Fig. S4 and Table S2, available at *JXB* online). In the following, the analysis concentrated on the major *QTL4-1.*

### A hotspot of recombination is detected in the intergenic interval between *Bx8* and *Bx5*

In order to determine whether *QTL4-1* comprised the *cis*-element(s) required for high 24 dai *Bx1* expression, RIL MO038 (Supplementary Table S3, available at *JXB* online) was crossed to B73 for fine mapping. MO038 had a late DIMBOA content that was more than twice the Mo17 level. With respect to *Bx1* expression, MO038 had the same properties as Mo17: the transcript level in 24 dai plants was as high as that in Mo17, allele-specific expression was displayed in the hybrid, and the transcript level in the hybrid was about 30% lower than the mid-parent value (Supplementary Fig. S5, available at *JXB* online). In contrast to Mo17, the RIL MO038 had homozygous B73 conformation for *QTL-1* and the largest part of *QTL4-2*. These two QTL regions will be homozygous in the F1 and all selfed progeny.

Two B73×MO038 hybrid plants were selfed and the progeny used for fine mapping in the interval between marker M210 (chromosome 4: 3 045 994–3 046 270) and M5 (chromosome 4: 3 250 998–3 251 489), which is the central region of *QTL4-1*. For the analysis, 11 co-dominant polymorphic markers were generated (Supplementary Table S4, available at *JXB* online). Twenty recombination events (Supplementary Fig. S6) were detected by screening of 750 plants representing 1500 gametes. Two hotspots of recombination were revealed. The borders are given by the markers M210 and M148 (seven recombination events within 62kb), and M148 and M137 (13 recombination events within 11kb) (Supplementary Fig. S6), respectively. No recombination was detected in the 132kb DNA stretch between markers M137 and M5. The majority of recombination was located close to (eight recombinations) or within (five recombinations) the region 3 112 492–3 116 365. This 3.9kb region was termed the ‘distal *cis*-element’ (DICE). It was present as a duplication in Mo17 (Supplementary Fig. S7A, available at *JXB* online). While one DICE copy in Mo17 was almost identical to that of B73, the other copy had several sequence alterations (Supplementary Fig. S7A, B). The structure of the DICE sequence in Mo17 was verified by Southern blot analysis (Supplementary Fig. S7C) and sequencing of the genomic and BAC DNA. The recombination rate in the DICE element was 74 cM Mb^–1^, which is almost 30 times higher than the genome’s average and even above the values generally found for genic regions (Dooner *et al.*, 1985). At present, it is unclear whether a specific sequence segment of DICE or its duplication in Mo17 triggers recombination in this region.

### Mo17-specific *cis*-elements required for prolonged and allele-specific *Bx1* transcription are located within 141kb upstream of the gene

**Fig. 4.**
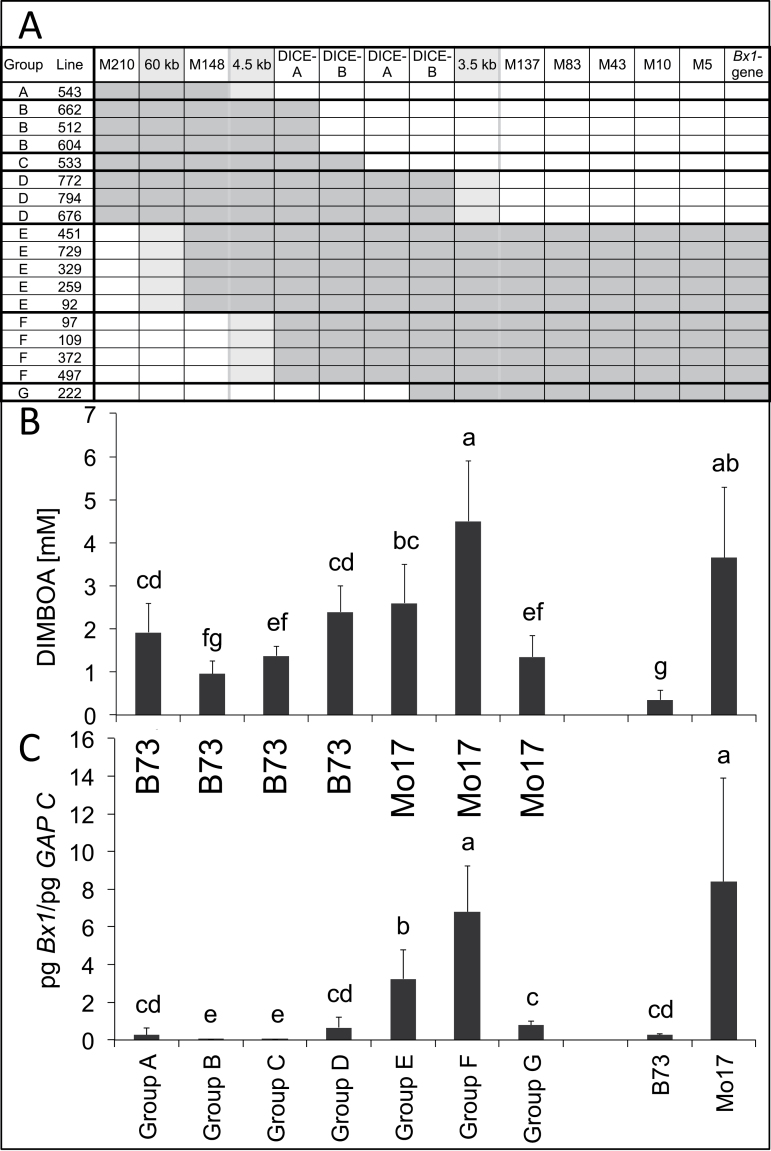
Analysis of recombinants. (A) Schematic presentation of recombination breakpoints. Marker positions are indicated in the header line and the distance of the marker to *Bx1* is indicated by the number, e.g. M137 is located 137kb upstream of *Bx1*. The size of gaps between the markers demonstrating recombination in the neighbourhood of DICE is given in the light grey fields. The DICE region is delimited by grey lines. Grey, Mo17 genotype, white, B73 genotype, light grey: unknown genotype. (B, C) DIMBOA content at 24 dai (B) and *Bx1* transcript level at 24 dai (C) in the respective groups of recombinants. All homozygous recombinants of group A–D were crossed with Mo17 and all recombinants of group E–G with B73, to yield heterozygous conformation for *Bx1*. B73 or Mo17 in (C) indicates whether the transcript level of the recombinant chromosome’s *Bx1* gene is the lower (B73-type) or the higher (Mo17-type) one. Statistical analysis was by Kruskal–Wallis test and multiple comparison of treatments. Mean values and standard deviation are indicated. Identical letters above columns indicate no statistical difference (*P*>0.5).

The recombinants made it possible to estimate the contribution of the sequences upstream and downstream of DICE, and the effect of the DICE duplication on late DIMBOA content, *Bx1* gene expression, and allele-specific expression. To generate homozygosity with respect to the *QTL4-1* region, the recombinants were selfed and the F3 genotyped (Fig. 4A). Either the homozygous F3 plants or F4 progeny thereof were analysed for DIMBOA content and *Bx1* transcript level. For allele-specific *Bx1* expression analysis, these lines were crossed with either Mo17 or B73 to generate heterozygosity with respect to *Bx1*. It was expected for the F3 and F4 material analysed that loci unlinked to *QTL4-1* would segregate independently. Recombinants with exchanges in the same region were grouped together in the following analyses (Fig. 4, groups A–G).

The DIMBOA content differed by a factor of 4.5 (Fig. 4B) in the homozygous recombinants. The high value of the parental MO038 line was not reached, but all lines had values higher than B73. The DIMBOA concentration tended to increase with the presence of the Mo17 genotype in and around the DICE region. The highest late DIMBOA content was detected in group F. The reciprocal exchange (group A) had reduced DIMBOA levels and a significant decrease was found for group G, which was distinguished from group F by loss of the Mo17 conformation of the DICE sequence (Fig. 4B).

The variation in the late *Bx1* expression level in the recombinants was more than 60-fold (Fig. 4C) and was not absolutely correlated with the DIMBOA content. The *Bx1* transcript level of Mo17 was reached by the recombinants that had both the Mo17 constitution for DICE and Mo17 sequences downstream thereof (group F). If the DICE duplication was missing (Fig. 4C, group G), the transcript level was significantly reduced. This result delimited upstream sequences influencing *Bx1* transcript level to the DICE sequence. However, presence of the duplication was not sufficient to confer high late *Bx1* mRNA levels if the downstream sequences came from the B73 genome (group D). In hybrids, the *Bx1* transcript level of the allele encoded by the recombinant chromosome was according to its genotype (groups A–D, B73-like, groups E–G, Mo17-like; Fig. 4). Hence, the recombination breakpoint closest to *Bx1* (DICE, 141kb upstream of *Bx1*) gave the upstream limit for localization of the *cis*-element(s) conferring allele-specific transcription. However, although in the hybrid B73 recombinant 222 (group G) the Mo17 allele of *Bx1* contributed by the recombinant had a higher transcript level than the B73 counterpart, the absolute *Bx1* transcript level was low compared with B73×Mo17 hybrids (Supplementary Fig. S8, available at *JXB* online). This implied that the Mo17 DICE sequence that was missing in recombinant 222, in conjunction with a further *cis*-element, was required for high 24 dai *Bx1* expression.

**Fig. 5.**
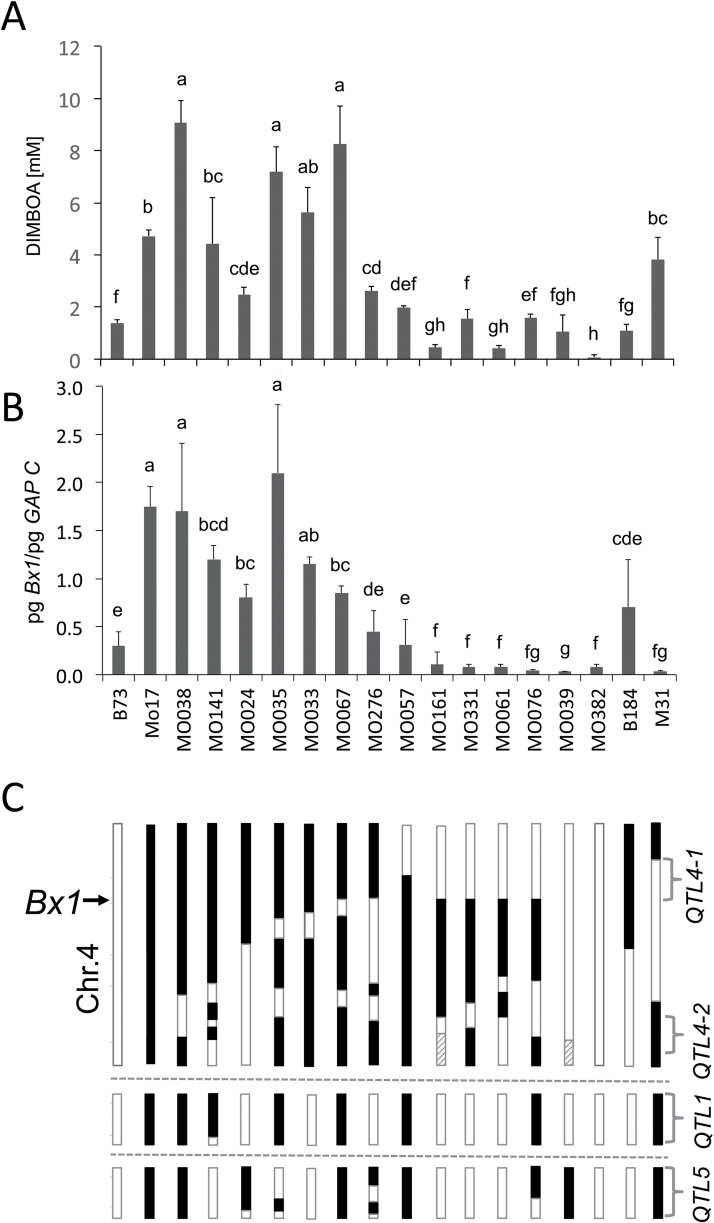
Analysis and genotypes of selected lines. (A, B) DIMBOA content (A) and *Bx1* mRNA level (B) in 24 dai plants. Statistical analysis was by Kruskal–Wallis test and multiple comparison of treatments. Mean values and standard deviation are indicated. Identical letters above columns indicate no statistical differences (*P*>0.5). (C) Schematic representation of the genotypes of these lines for chromosome 4 and the QTL regions on chromosomes 1 and 5. White, B73; black, Mo17; hatched, hybrid genotype. The positions of *Bx1* and of QTLs are indicated. For genotyping, the markers listed in Supplementary material were used. The position of the QTL is given in Supplementary Table S2.

To refine the mapping of the *cis*-elements, RILs were analysed (Fig. 5), which represent sets with opposing genotypes upstream of *Bx1* (set 1, Mo17 genotype: MO038, MO141, MO024, MO035, MO033, MO067, and MO276; set 2, B73 genotype: MO161, MO0331, MO061, MO076, MO039, and MO382) and harbour different proportions of the B73 genome downstream thereof (Fig. 5C). Set 1 RILs indicated that chromosome 4 sequences downstream of *Bx1* might also modulate *Bx1* transcript levels. With the exception of MO276, all of these lines had DIMBOA and *Bx1* levels that were significantly higher than B73 and DIMBOA levels and *Bx1* expression was correlated (Spearman correlation coefficient=0.75, *P*<0.001; Fig. 5A, B). By contrast, all lines of set 2 had low DIMBOA and low *Bx1* transcript levels. The data corroborated the impact of DICE and downstream sequences on *Bx1* expression detected in the recombinant lines.

Representative RILs and two B73×Mo17 NILs (Eichten *et al.*, 2011; see below) were crossed with Mo17 or B73 to generate progeny with heterozygous conformation for the *Bx1* gene. These hybrids were analysed for allele-specific expression (Supplementary Fig. S8). All sequences downstream of *Bx1* were present in the B73 conformation in NIL B184 and RIL MO276 (Fig. 5C). For both lines, allele specificity was detected. Therefore, the responsible *cis*-elements were located between DICE and the *Bx1* coding sequence. Allele-specific expression of all other lines was in accordance (Supplementary Fig. S8). In MO067 and MO276, *Bx1* had a B73 genotype and the upstream sequences were Mo17. In the hybrids generated by crossing with Mo17, both alleles were expressed at the same level. Hence, the *cis*-elements relevant for allele specificity were present in these RILs and no sequences within the gene itself were required. MO057 and the recombinant 222 (see above), which both had the recombination breakpoints within the DICE sequence leading to a monomeric hybrid DICE sequence, displayed allele-specific expression. This result revealed that it was the downstream sequences and not the duplication of the DICE sequence conferring allele specificity. The DICE sequence therefore mainly influences the *Bx1* transcript level, while allele specificity is mediated by additional *cis*-element(s) located in the DNA stretch of 141kb between DICE and *Bx1* in Mo17.

### High late DIMBOA levels can be established independent of the transcription of the biosynthetic genes

**Fig. 6.**
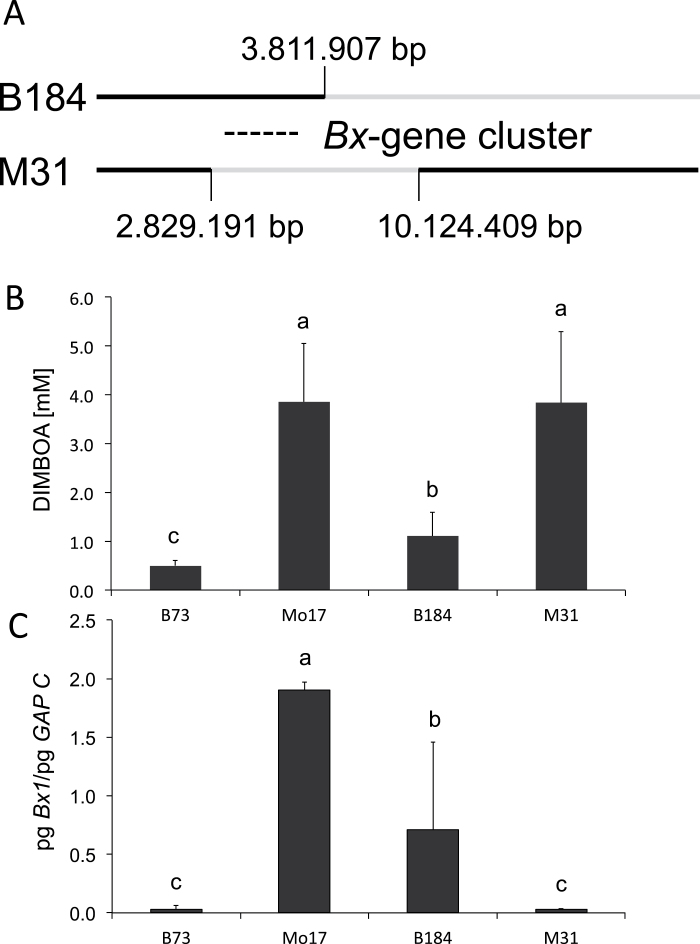
Analysis of NILs. (A) Scheme of the B73 isogenic line B183 and the Mo17 isogenic line M31. The Mo17 genotype is indicated in black and the B73 genotype in grey. The dashed line indicates the position of the clustered *Bx* genes (*Bx1*–*Bx5* and *Bx8*). (B, C) DIMBOA content (B) and *Bx1* transcript levels (C) in 24 dai plants. Statistical analysis was by Kruskal–Wallis test and multiple comparison of treatments. Mean values and standard deviation are indicated. Identical letters above columns indicate no statistical differences (*P*>0.5).

B73×Mo17 NILs were included in the study (Fig. 6). From the NIL collection, the lines B184 (B73 background) and M31 (Mo17 background) were chosen, which had Mo17 and B73 introgressions in the *QTL4-1* region (Fig. 6A). Surprisingly, M31, which had the B73 conformation for *QTL4-1* including the whole *Bx* gene cluster, had a high 24 dai DIMBOA content. The benzoxazinoid level in the reciprocal NIL B184 was increased relative to B73 but was significantly lower than in Mo17 and M31 (Fig. 6B). With respect to *Bx1* transcript levels, the NILs behaved as expected: M31 had levels as low as B73, and B184 had an increased *Bx1* transcript level compared with B73 (Fig. 6C). This showed that in Mo17 a mechanism exists that confers the prolonged presence of elevated DIMBOA concentrations independent of transcriptional activity of the *Bx1* gene. The finding was in line with the data for RILs and recombinants that showed no absolute correlation between *Bx1* mRNA levels and benzoxazinoid content.

### Influence of *trans*-acting factors on *Bx1* transcript levels

**Fig. 7.**
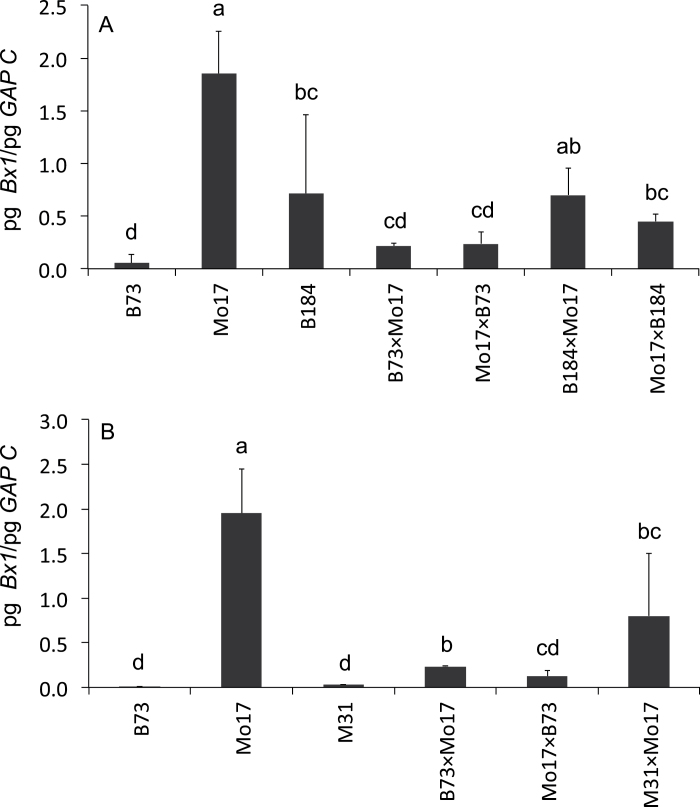
*Bx1* transcript levels in 24 dai hybrids of the NILs. (A) In the B184×Mo17 hybrid, *Bx1* is homozygous for the Mo17 allele. (B) In the M31×Mo17 hybrid, the Mo17 and B73 alleles are present. The transcript levels were normalized to *GAPC*. Statistical analysis was by Kruskal–Wallis test and multiple comparison of treatments. Mean values and standard deviation are indicated. Identical letters above columns indicate no statistical differences (*P*>0.5).

The previous analysis located *cis*-elements of *Bx1* gene transcription to the *QTL4-1* region. Transcriptional regulation is the result of the interaction between *cis*-elements and *trans*-acting factors. The finding that in B73×Mo17 hybrids the mid-parent value of the *Bx1* transcript level was not reached (e.g. Fig. 3D) could be explained by a dose-dependent activator(s) present in Mo17 and by a negative factor(s) in the B73 genome. Investigation of the NIL hybrids was carried out to reveal whether, in addition to the *cis*-elements, *trans*-factors were present in the *QTL 4-1* region (Fig. 7). B184×Mo17 hybrids had a homozygous Mo17 constitution at *QTL4-1* and were heterozygous for the rest of the genome. The *Bx1* transcript level was twice as high as in the B73×Mo17 hybrids but significantly lower than in Mo17 (Fig. 7A). The difference in transcript level compared with Mo17 had to be ascribed to *trans*-factors that are not located in the Mo17 region of B184 (position 0 to 3 811 907 on chromosome 4). Likewise, analysis of M31×Mo17 hybrids excluded major *trans*-factors in the region between 2 818 191 and 10 124 409bp on chromosome 4, and the B73 contribution was restricted to this introgressed sequence. M31×Mo17 and B73×Mo17 hybrids were both heterozygous for the *Bx1* to DICE region, but the *Bx1* transcript level in M31×Mo17 was increased (Fig. 7B), implying a balance of negative and positive *trans*-factors similar to Mo17 in the NIL hybrid.

The Mo17 haplotype might be required for productive *trans*-factor binding to the *cis*-elements. In this case, only RILs that have the Mo17 genotype in the DICE to *Bx1* region would be informative for mapping of the *trans*-factors. The respective population of IBM302 was employed for QTL mapping based on 24 dai DIMBOA content. Three QTLs with LOD scores above 4.8 were detected (Supplementary Fig. S10, available at *JXB* online). None of these overlapped with the minor QTL on chromosomes 1, 4, and 5 found in the analysis of the IBM population. Interestingly, the QTL on chromosome 5 overlapped with a major QTL for DIMBOA content detected in a study by Betsiashvili *et al.* (2015). However, these QTLs did not obviously correlate with *Bx1* expression in the RILs (Supplementary Fig. S11, available at *JXB* online), and no information about *trans*-factors involved in 24 dai *Bx1* expression was retrieved. A QTL for variation of DIMBOA content on chromosome 1 revealed by Meihls *et al.* (2013) using B73×CML322 RILs (bin 1.04) did not coincide with the QTL found here (bin 1.06).

### *Bx2* transcript level is not influenced by *cis*-elements

The *Bx* genes form a biosynthetic cluster. It has been speculated that clustering of the *Bx* genes favours co-ordinated regulation (Gierl and Frey, 2001). Co-ordinate regulation by shared elements was expected especially for *Bx1* and *Bx2* since both genes are separated by only 2.5kb. Late *Bx2* levels for B73 and Mo17 were not significantly different, but this did not exclude the possibility that allele specificity exists. There was no sequence difference for the two *Bx2* alleles that would allow differentiation of expression by RT-qPCR. Instead, an analysis by cDNA sequencing was performed. Three SNPs were analysed for *Bx1* and four for *Bx2*. The predominant expression of the Mo17 allele of *Bx*1 was obvious (Supplementary Fig. S9, available at *JXB* online). In contrast, both *Bx2* alleles were expressed at the same level. The *cis*-elements defined in the *Bx1* upstream region hence were not effective for *Bx2* expression.

### Increased *Bx1* expression is sufficient to increase DIMBOA content in older plants

**Fig. 8.**
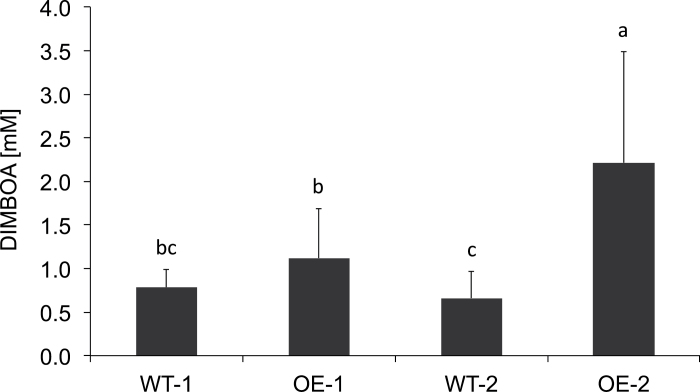
DIMBOA content in 24 dai transgenic plants overexpressing *Bx1*. Statistical analysis was by Kruskal–Wallis test and multiple comparison of treatments. Mean values and standard deviation are indicated. Identical letters above columns indicate no statistical differences (*P*>0.5).

The previous analyses revealed a major QTL for high late benzoxazinoid concentration in the region of the *Bx* gene cluster and further analysis showed that the main feature correlated with *QTL4-1* was elevated late *Bx1* expression mediated by *cis*-elements. However, the investigations demonstrated that further mechanisms exist that result in high late DIMBOA concentrations and are independent of *Bx1* transcription. To assay whether prolonged *Bx1* gene expression was sufficient to elevate the benzoxazinoid content in older plants, we attempted to increase *Bx1* expression by transgenic expression of the gene driven by the ubiquitin promoter. The hybrid maize line HiII (Zhao *et al.*, 1998) was used for transformation with *Agrobacterium tumefaciens* (Frame *et al.*, 2002). There was no obvious phenotypic difference between transgenic plants and the non-transformed siblings at the seedling and adult stage. In seedlings, the amount of *Bx1* mRNA detected by qRT-PCR in HiII was similar to B73 (data not shown). In transgenic progeny, the *Bx1* transcript levels consistently exceeded glyceraldehyde 3-phosphate dehydrogenase values at 24 dai and were more than 20-fold increased compared with the non-transgenic sibling plants (Supplementary Table S5, available at *JXB* online). No correlation of transgenic *Bx1* expression and transcript levels of the other *Bx* genes was detected (Supplementary Table S5). Segregating T1 progeny were analysed for benzoxazinoids at 24 dai. Individuals with the transgenic *Bx1* allele had DIMBOA concentrations of up to 3.9mM and could be considered high lines. The average value reached in the transgenics was 1.8mM, which was significantly higher than the level of 0.7mM displayed in average by the non-transgenic sibling plants (Fig. 8). Prolonged *Bx1* expression, as detected in Mo17, therefore seems to be an effective mechanism to sustain effective benzoxazinoid concentrations during the later stages of plant development.

## Discussion

### Transcriptional regulation of *Bx1* affects DIMBOA concentration

The starting point of the study was the survey of genetic variability in DIMBOA content in maize beyond the seedling stage (24 dai). In the NAM panel, which represents 80% of the variability in maize, prolonged presence of the benzoxazinoid was displayed by three out of 26 lines; among these, Mo17 was prominent (Fig. 1). B73 belongs to the majority of lines with low DIMBOA level at 24 dai and therefore the IBM302 mapping population offers a solid basis for QTL mapping. The major QTL for DIMBOA content in 24 dai plants mapped to the cluster of *Bx* genes (*QTL4-1*; Supplementary Fig. S4). A QTL in this position has been determined in different studies aiming directly (Butrón *et al.*, 2010) or indirectly via the impact of benzoxazinoids on insect resistance (Jampatong *et al.*, 2002; Cardinal *et al.*, 2006; Betsiashvili *et al.*, 2015) to detect loci influencing DIMBOA concentration. Mo17 was also exceptional within the NAM diversity panel with respect to high *Bx1* transcript levels in 24 dai plants. The genetic analysis demonstrated that high *Bx1* transcript levels were correlated with relatively high DIMBOA concentrations at later developmental stages (24 dai) of the maize plant. The alteration of the *Bx* gene transcription pattern was limited to *Bx1*; the pattern for all other *Bx* genes was similar for Mo17 and B73 (Supplementary Fig. S2). The increase in DIMBOA content in 24 dai plants by transgenic overexpression of *Bx1* also indicated that prolonged *Bx1* transcription was sufficient to generate elevated DIMBOA levels beyond the seedling stage. Furthermore, the overexpressing plants demonstrated that the correlation between *Bx1* mRNA level and DIMBOA concentration in the older plant was not restricted to the Mo17 genetic background. Hence, it can be speculated that BX1, the branch-point enzyme of the biosynthetic pathway, has a bottleneck function in DIMBOA biosynthesis of maturing plants.

This finding is in line with the result of a recent candidate association analysis investigating the correlation of *Bx* gene sequence polymorphisms on DIMBOA content in older (32 dai) plants (Butrón *et al.*, 2010). A significant association was determined for *Bx1* sequence polymorphisms but for none of the other *Bx* genes.

### Late *Bx1* transcription depends on DICE

In the candidate gene association study by Butrón *et al.* (2010), the causal polymorphism connected to *Bx1* in the QTL for DIMBOA content could not be detected. The analysis was restricted to the gene sequence and proximal regions. Fine mapping of the IBM302 *QTL4-1* in our study included 250kb of the *Bx1* upstream region and revealed the sequence DICE as required for increased *Bx1* transcript levels at 24 dai. The DICE element is located 141kb upstream of *Bx1* and the conformation that promotes *Bx1* transcription in Mo17 is a tandem duplicate. This is reminiscent of the long-range effect of the upstream approximately 107kb regulatory elements of the *b1* gene of maize, which is dependent on multimerization of the sequence element. Here, an impact of the multimers on binding of regulatory proteins (Brzeska *et al.*, 2010) and formation of regulatory small RNA (Belele *et al.*, 2013) was hypothesized. At present, it is unknown how the increase in *Bx1* transcript level is mediated by the Mo17 haplotype. In addition to DICE, further *cis*-element(s) that influence *Bx1* transcript levels have been delimited to the region between the *Bx1* gene and DICE. These sequences are responsible for the allele-specific expression of *Bx1*. No recombinants that would allow more precise positioning of the additional *cis*-elements were available from the fine mapping and IBM302 RILs.

Strikingly, allele-specific and increased transcription was restricted to *Bx1* and included neither *Bx2*, which is located only 2.5kb downstream, nor any other *Bx* gene in the cluster (Supplementary Fig. S9). In hybrids, both *Bx2* alleles were expressed at the same level in seedlings and late plants. Allele specificity could not be analysed for *Bx5* and *Bx8* since polymorphisms are lacking, but both genes, which flank DICE (Fig. 1), were expressed at comparable low rates in Mo17 and B73 24 dai plants (Supplementary Fig. S2). This was unexpected since *Bx5* is in close proximity to DICE (0.5kb), and *Bx8* is located between DICE and *Bx1*. These results demonstrated that clustered genes are not necessarily co-regulated and rather indicates an individual regulation, in this case of the gene encoding the signature enzyme of the pathway.

A possible contribution of *trans*-factors located within the *QTL4-1* region was investigated by analysis of hybrid NIL lines. The results excluded major *trans*-factors in this genomic region (Fig. 7). The minor QTLs *QTL3*, *QTL4-2*, and *QTL5* might coincide with *trans*-factors. The genotypes of the IBM302 RILs analysed in detail (Fig. 5), however, showed no correlation of the genotype in the minor QTL and 24 dai *Bx1* transcript levels.

### High DIMBOA content at 24 dai is not strictly dependent on high *Bx1* transcript levels

DIMBOA analysis of the NAM panel lines demonstrated that high 24 dai DIMBOA levels could also occur when *Bx1* transcription was low. An analogous result was displayed for the NIL M31. M31 is of Mo17 genotype with the exception of the region around *QTL4-1*, which confers a low *Bx1* mRNA level at 24 dai due to the B73 genotype of DICE and downstream sequences; nevertheless, M31 has an extremely high DIMBOA content (Fig. 6). Similar findings of unexpectedly high DIMBOA levels in the presence of the B73 genotype in the *Bx* gene region were reported in a recent study (Betsiashvili *et al.*, 2015) on correlation between aphid control and DIMBOA content using analogous NILs from the same collection of lines (Eichten *et al.*, 2011). Since benzoxazinoid steady-state levels are the product of formation, storage, and decay, other processes in addition to *Bx1* transcription, can cause high DIMBOA levels in 24 dai maize plants.

### The *Bx* gene cluster contains hotspots of recombination

The DIMBOA biosynthetic genes in maize are organized as a cluster. The core harbours six *Bx* genes and spans 264kb of genomic DNA (Fig. 1). In maize, this is considered a gene-rich region in relation to the 2.3 Gb genome (Schnable *et al.*, 2009). Recombination frequencies are generally higher in unique, mostly genic regions compared with regions with repetitive sequences (Yao *et al.*, 2002; Dooner and He, 2008). Several hotspots of recombination have been detected by the fine mapping within the 3.9kb DICE element and in the adjacent genomic regions (Fig. 4 and Supplementary Fig. S6). The recombination rate in the DICE element is significantly higher than expected for genic regions. DICE is a unique sequence that is flanked by the *Bx5* gene and a long stretch of low repetitive sequence (MIPS annotation of repetitive sequence motifs, Kurtz *et al.*, 2008; MaizeGDB browser: http://www.maizegdb.org/gbrowse.php). DICE and the adjacent downstream genomic regions are hypomethylated in the third leaf of 18-d-old plants (Eichten *et al.*, 2011; MaizeGDB browser: http://www.maizegdb.org/gbrowse.php), indicating open chromatin conformation, as expected for recombinational hyperactive chromosomal regions (Goodstadt and Ponting, 2011).

Clustering of genes for secondary or specialized metabolic pathways is common in plants (reviewed by Osbourn, 2010). It was speculated that one of the evolutionary forces for the formation and maintenance of gene clusters is the suppression of recombination by close physical linkage of the genes. Thereby, gene clusters might have a selective advantage since superior allelic combinations are inherited preferentially once established in the coupling phase (Frey *et al.*, 2009; Field and Osbourn, 2012; Takos and Rook, 2012). The finding of recombination hotspots in the *Bx* gene cluster revealed this hypothesis in a different light. Also, transcriptional co-regulation of the clustered genes, a further hypothesis, does not apply to our findings.

We demonstrated allelic variation of *Bx1* gene expression and DIMBOA content at the 24 dai stage of leaf development for B73 and Mo17 and detected a recombination hotspot connected with a *cis*-element involved in allele-specific transcription. A direct correlation between a recombination hotspot and variation of allelic expression has been demonstrated for eight genes in the *Sh1*–*Bz1* chromosomal region of maize (Hawkins *et al.*, 2014) and seems to be a more common feature. The high recombination frequency in the *Bx* gene cluster is consistent with the hypothesis that diversity in defence gene expression should be beneficial at the population level. It would be surprising if there were only a small number of optimal haplotypes, given the wide variety of environments in which maize (or teosinte) grows. This does not seem to be inconsistent with the hypothesis that it is beneficial to have a genetically linked gene cluster that can be inherited as a biosynthetic unit.

### Potential to increase maize protection by elevated DIMBOA concentration at later growth stages

The analyses revealed that high *Bx1* transcript levels in older plants prolonged the presence of effective DIMBOA concentrations. Several IBM302 RIL lines, e.g. MO038, had significantly higher 24 dai DIMBOA content than Mo17. All of these lines had the Mo17 haplotype for the DICE and the downstream *cis*-element region. The values for MO038 (Supplementary Fig. S5) demonstrated that the higher DIMBOA content compared with Mo17 was not caused by a further increase in *Bx1* transcript level but rather by independent mechanisms. This offers the possibility of using high late *Bx1*-expressing lines and breeding on this background to further increase the long-lasting protection by DIMBOA.

The feature of high late DIMBOA content was relatively rare among the 26 NAM panel lines. Only three had a significant DIMBOA concentration at 24 dai. The NAM lines reflect a certain amount of manmade selection. Besides representation of large variation, the NAM lines were chosen to include public lines of importance to temperate breeding and important tropical and subtropical lines (Liu *et al.*, 2003), and inbreds were selected that produce seed in the summer in the USA (Yu *et al.*, 2008). This could have reduced the time interval for DIMBOA biosynthesis for the benefit of increased yield or other agronomic traits. It is assumed that plant defence, especially constitutive mechanisms like the preformed toxic benzoxazinoids of the grasses and glucosinolates of the Brassicaceae, are costly by detracting resources from growth and reproduction (Herms and Mattson, 1992). Analysis in *Arabidopsis thaliana* using mutants revealed that glucosinolate biosynthesis indeed evokes growth costs (Züst *et al.*, 2011). The cost in biosynthesis might render high late DIMBOA levels beneficial only in certain environments.

## Supplementary data

Supplementary data are available at *JXB* online.

Supplementary Fig. S1. DIMBOA concentration of seedlings of the NAM panel lines.

Supplementary Fig. S2. *Bx* gene transcript levels of B73 and Mo17.

Supplementary Fig. S3. DIMBOA concentration in the IBM302 RILs.

Supplementary Fig. S4. QTL analysis for DIMBOA.

Supplementary Fig. S5. Characterization of the RIL MO038.

Supplementary Fig. S6. Genotype of recombinants.

Supplementary Fig. S7. Characterization of the distal *cis*-element (DICE).

Supplementary Fig. S8. Analysis of *Bx1* transcript levels in 24 dai heterozygous plants.

Supplementary Fig. S9. Analysis of *Bx1* and *Bx2* allele-specific expression.

Supplementary Fig. S10. QTL analysis in IBM302 subset.

Supplementary Fig. S11. *Bx1* expression analysis and genotype of selected IBM lines.

Supplementary Table S1. Primer and PCR conditions.

Supplementary Table S2. Composite interval mapping.

Supplementary Table S3. MO038 genotype.

Supplementary Table S4. Marker for fine mapping.

Supplementary Table S5. *Bx* gene transcript levels in transgenics at 24 dai.

**Supplementary material.** List of markers.

Supplementary Data

## Abbreviations:

BAC: bacterial artificial chromosome
dai: days after imbibition
DIBOA: 2,4-dihydroxy-1,4-benzoxazin-3-one
DIMBOA: 2,4-dihydroxy-7-methoxy-1,4-benzoxazin-3-one
GAPC: cytosolic glyceraldehyde 3-phosphate dehydrogenase
HPLC: high-performance liquid chromatography
NAM: maize nested association mapping
NIL: near-isogenic line
QTL: quantitative trait locus
RIL: recombinant inbred line
SNP: single-nucleotide polymorphism.

## References

[CIT0001] Basten CJ Weir B Zeng ZB 1997 QTL Cartographer: a reference manual and tutorial for QTL mapping. Department of Statistics, North Carolina State University: Raleigh, NC http://statgen.ncsu.edu/qtlcart/manual/.

[CIT0002] Belele CL Sidorenko L Stam M Bader R Arteaga-Vazquez MA Chandler VL 2013 Specific tandem repeats are sufficient for paramutation-induced *trans*-generational silencing. PLoS Genetics 9, e1003773. 24146624 10.1371/journal.pgen.1003773 PMC3798267

[CIT0003] Betsiashvili M Ahern KR Jander G 2015 Two *Rhopalosiphum maidis* (corn leaf aphid) resistance loci in maize inbred line Mo17 have additive effect. Journal of Experimental Botany 66, 571–578. 25249072 10.1093/jxb/eru379 PMC4286405

[CIT0004] Brzeska K Brzeski J Smith J Chandler VL 2010 Transgenic expression of CBBP, a CXC domain protein, establishes paramutation in maize. Proceedings of the National Academy of Sciences, USA 107, 5516–5521. 10.1073/pnas.1001576107 PMC2851796 20212123

[CIT0005] Butrón A Chen YC Rottinghaus GE McMullen MD 2010 Genetic variation at *bx1* controls DIMBOA content in maize. Theoretical and Applied Genetics 120, 721–734. 19911162 10.1007/s00122-009-1192-1

[CIT0006] Campos F Atkinson J Arnason JT Philogene BJR Morand P Werstiuk NH Timmins G 1989 Toxicokinetics of 2,4-dihydroxy-7-methoxy-1,4-benzoxazin-3-one (DIMBOA) in the European corn borer, *Ostrinia nubilalis* (Hübner). Journal of Chemical Ecology 15, 1989–2001. 24272290 10.1007/BF01207432

[CIT0007] Cardinal AJ Lee M Guthrie WD Bing J Austin DF Leldboom LR Senior ML 2006 Mapping of factors for resistance to leaf-blade feeding by European corn borer (*Ostrinia nubilalis*) in maize. Maydica 51, 93–102.

[CIT0008] Christensen AH Quail PH 1996 Ubiquitin promoter-based vectors for high-level expression of selectable and/or screenable marker genes in monocotyledonous plants. Transgenic Research 5, 213–218. 8673150 10.1007/BF01969712

[CIT0009] Clark RM Wagler TN Quijada P Doebley J 2006 A distant upstream enhancer at the maize domestication gene *tb1* has pleiotropic effects on plant and inflorescent architecture. Nature Genetics 38, 594–597. 16642024 10.1038/ng1784

[CIT0010] Dafoe NJ Thomas JD Shirk PD Legaspi ME Vaughan MM Huffaker A Teal PE Schmelz EA 2013 European corn borer (*Ostrinia nubilalis*) induced responses enhance susceptibility in maize. PLoS One 8, e73394. 24023868 10.1371/journal.pone.0073394 PMC3759431

[CIT0011] Dooner HK He L 2008 Maize genome structure variation: interplay between retrotransposon polymorphisms and genic recombination. The Plant Cell 20, 249–258. 18296625 10.1105/tpc.107.057596 PMC2276454

[CIT0012] Dooner HK Weck E Adams S Ralston E Favreau M English J 1985 A molecular genetic analysis of insertions in the bronze locus in maize. Molecular and General Genetics 200, 240–246.

[CIT0013] Eichten SR Swanson-Wagner RA Schnable JC 2011 Heritable epigenetic variation among maize inbreds. PLoS Genetics 7, e1002372. 22125494 10.1371/journal.pgen.1002372 PMC3219600

[CIT0014] Field B Osbourn A 2012 Order in the playground. Formation of plant gene clusters in dynamic chromosomal regions. Mobile Genetic Elements 2, 46–50. 22754752 10.4161/mge.19348 PMC3383449

[CIT0015] Frame BR Shou H Chikwamba RK 2002 *Agrobacterium tumefaciens*-mediated transformation of maize embryos using a standard binary vector system. Plant Physiology 129, 13–22. 12011333 10.1104/pp.000653 PMC1540222

[CIT0016] Frey M Chomet P Glawischnig E 1997 Analysis of a chemical plant defense mechanism in grasses. Science 277, 696–699. 9235894 10.1126/science.277.5326.696

[CIT0017] Frey M Huber K Park WJ Sicker D Lindberg P Meeley RB Simmons CR Yalpani N Gierl A 2003 A 2-oxoglutarate-dependent dioxygenase is integrated in DIMBOA-biosynthesis. Phytochemistry 62, 371–376. 12620350 10.1016/s0031-9422(02)00556-3

[CIT0018] Frey M Schullehner K Dick R Fiesselmann A Gierl A 2009 Benzoxazinoid biosynthesis, a model for evolution of secondary metabolic pathways in plants. Phytochemistry 70, 1645–1651. 19577780 10.1016/j.phytochem.2009.05.012

[CIT0019] Gierl A Frey M 2001 Evolution of benzoxazinone biosynthesis and indole production in maize. Planta 213, 493–498. 11556781 10.1007/s004250100594

[CIT0020] Goodstadt L Ponting CP 2011 Is the control of recombination conserved among diverse eukaryotes? Heredity 106, 710–711. 20606688 10.1038/hdy.2010.88 PMC3186235

[CIT0021] Grombacher AW Russell WA Guthrie WD 1989 Resistance to first-generation European corn borer (Lepidoptera: Pyralidae) and DIMBOA concentration in midwhorl leaves of the BS9 maize synthetic. Journal of the Kansas Entomological Society 62, 103–107.

[CIT0022] Grün S Frey M Gierl A 2005 Evolution of the indole alkaloid biosynthesis in the genus *Hordeum*: distribution of gramine and DIBOA and isolation of the benzoxazinoid biosynthesis genes from *Hordeum lechleri* . Phytochemistry 66, 1264–1272. 15907959 10.1016/j.phytochem.2005.01.024

[CIT0023] Hawkins JS Delgado V Feng L Carlise M Dooner HK Bennetzen JL 2014 Variation in allelic expression associated with a recombination hotspot in *Zea mays* . The Plant Journal 79, 375–384. 24761964 10.1111/tpj.12537

[CIT0024] Herms DA Mattson WJ 1992 The dilemma of plants: to grow or defend. Quarterly Review of Biology 67, 283–335.

[CIT0025] Huffaker A Pearce G Veyrat N 2013 Plant elicitor peptides are conserved signals regulating direct and indirect antiherbivore defense. Proceedings of the National Academy of Sciences, USA 110, 5707–5712. 10.1073/pnas.1214668110 PMC3619339 23509266

[CIT0026] Jampatong C McMullen MD Barry BD Darrah LL Byrne PF Kross H 2002 Quantitative loci for first- and second-generation European corn borer resistance derived from the maize inbred Mo47. Crop Science 42, 584–593.

[CIT0027] Jansen RC Stam P 1994 High resolution mapping of quantitative traits into multiple loci via interval mapping. Genetics 136, 1447–1455. 8013917 10.1093/genetics/136.4.1447 PMC1205923

[CIT0028] Jonczyk R Schmidt H Osterrieder A 2008 Elucidation of the final reactions of DIMBOA-glucoside biosynthesis in maize: characterization of *Bx6* and *Bx7* . Plant Physiology 146, 1053–1063. 18192444 10.1104/pp.107.111237 PMC2259038

[CIT0029] King AJ Brown GD Gilday AD Larson TR Graham IA 2014 Production of bioactive diterpenoids in the Euphorbiaceae depends on evolutionarily conserved gene clusters. The Plant Cell 26, 3286–3298. 25172144 10.1105/tpc.114.129668 PMC4371829

[CIT0030] Klun JA Guthrie WD Hallauer AR Russell WA 1970 Genetic nature of the concentration of 2,4-dihydroxy-7-methoxy-2*H*-1,4-benzoxazin-3(4*H*)-one and resistance to the European corn borer in a diallel set of eleven maize inbreds. Crop Science 10, 87–90.

[CIT0031] Kurtz S Narechania A Stein JC Ware D 2008 A new method to compute K-mer frequencies and its application to annotate large repetitive plant genomes. BMC Genomics 9, 517. 18976482 10.1186/1471-2164-9-517 PMC2613927

[CIT0032] Lee M Sharopova N Beavis WD Grant D Katt M Blair D Hallauer A 2002 Expanding the genetic map of maize with the intermated B73×Mo17 (IBM) population. Plant Molecular Biology 48, 453–461. 11999829 10.1023/a:1014893521186

[CIT0033] Liu K. Goodman M. Muse S. Smith J.S. Buckler E. Doebley J 2003 Genetic structure and diversity among maize inbred lines as inferred from DNA microsatellites. Genetics 165, 2117–2128. 14704191 10.1093/genetics/165.4.2117 PMC1462894

[CIT0034] Long BJ Dunn GM Routley DG 1975 Relationship of hydroxamic acid content in maize and resistance to northern corn leaf blight. Crop Science 15, 333–335.

[CIT0035] Long BJ Dunn GM Bowman JS Routley DG 1977 Relationship of hydroxamic acid content in corn and resistance to the corn leaf aphid. Crop Science 17, 55–58.

[CIT0036] Monaco MK Taner ZS Palitha DD 2013 Maize metabolic network construction and transcriptome analysis. Plant Genome 6, doi:10.3835/plantgenome2012.09.0025.

[CIT0037] Meihls LN Handrick V Glauser G 2013 Natural variation in maize aphid resistance is associated with 2,4-dihydroxy-7-methoxy-1,4-benzoxazin-3-one glucoside methyltransferase activity. The Plant Cell 25, 2341–2355. 23898034 10.1105/tpc.113.112409 PMC3723630

[CIT0038] Niemeyer HM 1988 Hydroxamic acids (4-hydroxy-1,4-benzoxazin-3-ones), defence chemicals in the Gramineae. Phytochemistry 27, 3349–3358.

[CIT0039] Niemeyer HM 2009 Hydroxamic acids derived from 2-hydroxy-2*H*-1,4-benzoxazin-3(4*H*)-one: key defense chemicals of cereals. Journal of Agricultural and Food Chemistry 57, 1677–1696. 19199602 10.1021/jf8034034

[CIT0040] Nomura T Ishihara A Imaishi H Ohkawa H Endo TR Iwamura H 2003 Rearrangement of the genes for the biosynthesis of benzoxazinones in the evolution of Triticeae species. Planta 217, 776–782. 12734755 10.1007/s00425-003-1040-5

[CIT0041] Nomura T Nasuda S Kawaura K Ogihara Y Kato N Sato F Kojima T Toyoda A Iwamura H Endo TR 2008 Structures of the three homoeologous loci of wheat benzoxazinone biosynthetic genes *TaBx3* and *TaBx4* and characterization of their promoter sequences. Theoretical and Applied Genetics 116, 373–381. 18040657 10.1007/s00122-007-0675-1

[CIT0042] Nützmann HW Osbourn A 2014 Gene clustering in plant specialized metabolism. Current Opinion in Biotechnology 26, 91–99. 24679264 10.1016/j.copbio.2013.10.009

[CIT0043] Okada A Okada K Miyamoto K Koga J Shibuya N Nojiri H Yamane H 2009 OsTGAP1, a bZIP transcription factor, coordinately regulates the inductive production of diterpenoid phytoalexins in rice. Journal of Biological Chemistry 284, 26510–26518. 19635799 10.1074/jbc.M109.036871 PMC2785339

[CIT0044] Osbourn A 2010 Gene clusters for secondary metabolic pathways: an emerging theme in plant biology. Plant Physiology 154, 531–535. 20921179 10.1104/pp.110.161315 PMC2949040

[CIT0045] Salvi S Sponza G Morgante M 2007 Conserved noncoding genomic sequences associated with a flowering-time quantitative trait locus in maize. Proceedings of the National Academy of Sciences, USA 104, 11376–11381. 10.1073/pnas.0704145104 PMC2040906 17595297

[CIT0046] Schaeffer ML Harper LC Gardiner JM Andorf CM Campbell DA Cannon EK Sen TZ Lawrence CJ 2011 MaizeGDB: curation and outreach go hand-in-hand. Database 2011 : Bar022. 10.1093/database/bar022 PMC3104940 21624896

[CIT0047] Schnable PS Ware D Fulton RS 2009 The B73 maize genome: complexity, diversity, and dynamics. Science 326, 1112–1115. 19965430 10.1126/science.1178534

[CIT0048] Schullehner K Dick R Vitzthum F Schwab W Brandt W Frey M Gierl A 2008 Benzoxazinoid biosynthesis in dicot plants. Phytochemistry 69, 2668–2677. 18929374 10.1016/j.phytochem.2008.08.023

[CIT0049] Sicker D Frey M Schulz M Gierl A 2000 Role of natural benzoxazinones in the survival strategy of plants. International Review of Cytology 198, 319–346. 10804466 10.1016/s0074-7696(00)98008-2

[CIT0050] Stam M Belele C Dorweiler JE Chandler VL 2002 Differential chromatin structure within a tandem array 100kb upstream of the maize *b1* locus is associated with paramutation. Genes & Development 16, 1906–1918. 12154122 10.1101/gad.1006702 PMC186425

[CIT0051] Takos AM Rook F 2012 Why biosynthetic genes for chemical defense compounds cluster. Trends in Plant Science 17, 383–387. 22609284 10.1016/j.tplants.2012.04.004

[CIT0052] Virtanen AI Hietala PK 1955 2(3)-Benzoxazolinone, an anti-fusarium factor in rye seedlings. Acta Chemica Scandinavica 09, 1543.

[CIT0053] von Rad U Hüttl R Lottspeich F Gierl A Frey M 2001 Two glucosyltransferases are involved in detoxification of benzoxazinoids in maize. The Plant Journal 28, 633–642. 11851909 10.1046/j.1365-313x.2001.01161.x

[CIT0054] Wahlroos Ö Virtanen A 1959 Precursors of 6-methoxybenzoxazolinone in maize and wheat plants, their isolation and some of their properties. Acta Chemica Scandinavica 13, 1906–1908.

[CIT0055] Wegel E Koumproglou R Shaw and Osbourn A 2009 Cell type-specific chromatin decondensation of a metabolic gene cluster in oats. The Plant Cell 21, 3926–3936 20040536 10.1105/tpc.109.072124 PMC2814510

[CIT0056] Yao H Zhou Q Li J Smith H Yandeau M Nikolau BJ Schnable PS 2002 Molecular characterization of meiotic recombination across the 140-kb multigenic *a1-sh2* interval of maize. Proceedings of the National Academy of Sciences, USA 99, 6157–6162. 10.1073/pnas.082562199 PMC122919 11959909

[CIT0057] Yu J Holland JB McMullen MD Buckler ES 2008 Genetic design and statistical power of nested association mapping in maize. Genetics 178, 539–551. 18202393 10.1534/genetics.107.074245 PMC2206100

[CIT0058] Zeng ZB 1994 Precision mapping of quantitative trait loci. Genetics 136, 1457–1468. 8013918 10.1093/genetics/136.4.1457 PMC1205924

[CIT0059] Zhao ZY Gu W Cai T Tagliani LA Hondred DA Bond D Krell S Rudert ML Bruce WB Pierce DA 1998 Molecular analysis of T0 plants transformed by *Agrobacterium* and comparison of *Agrobacterium*-mediated transformation with bombardment transformation in maize. Maize Genetics Cooperation Newsletter 72, 34–37.

[CIT0060] Züst T Joseph B Shimizu KK Kliebenstein DJ Turnbull LA 2011 Using knockout mutants to reveal the growth costs of defensive traits. Proceedings of the Royal Society B: Biological Sciences 278, 2598–2526. 10.1098/rspb.2010.2475 PMC3136827 21270041

