# Supplementary material for: Prolonged expression of the BX1 signature enzyme is associated with a recombination hotspot in the benzoxazinoid gene cluster in Zea mays
Source: J Exp Bot. 2015 May 11;66(13):3917–30. doi: 10.1093/jxb/erv192 (PMC4473990; doi:10.1093/jxb/erv192)
Supplement: Supplementary Data [file supp_66_13_3917__index.html]

Prolonged expression of the BX1 signature enzyme is associated with a recombination hotspot in the benzoxazinoid gene cluster in Zea mays — Prolonged expression of the BX1 signature enzyme is associated with a recombination hotspot in the benzoxazinoid gene cluster in Zea mays — Supplementary Data 

# Prolonged expression of the BX1 signature enzyme is associated with a recombination hotspot in the benzoxazinoid gene cluster in *Zea mays*

## Supplementary Data

Data files

**Files in this Data Supplement:**

- Supplementary Data - Supplementary Data
