# Supplementary material for: Prolonged expression of the BX1 signature enzyme is associated with a recombination hotspot in the benzoxazinoid gene cluster in Zea mays
Source: J Exp Bot. 2015 May 11;66(13):3917–30. doi: 10.1093/jxb/erv192 (PMC4473990; doi:10.1093/jxb/erv192)
Supplement: Supplementary Data [file supp_erv192_jexbot138347_file001.pdf]

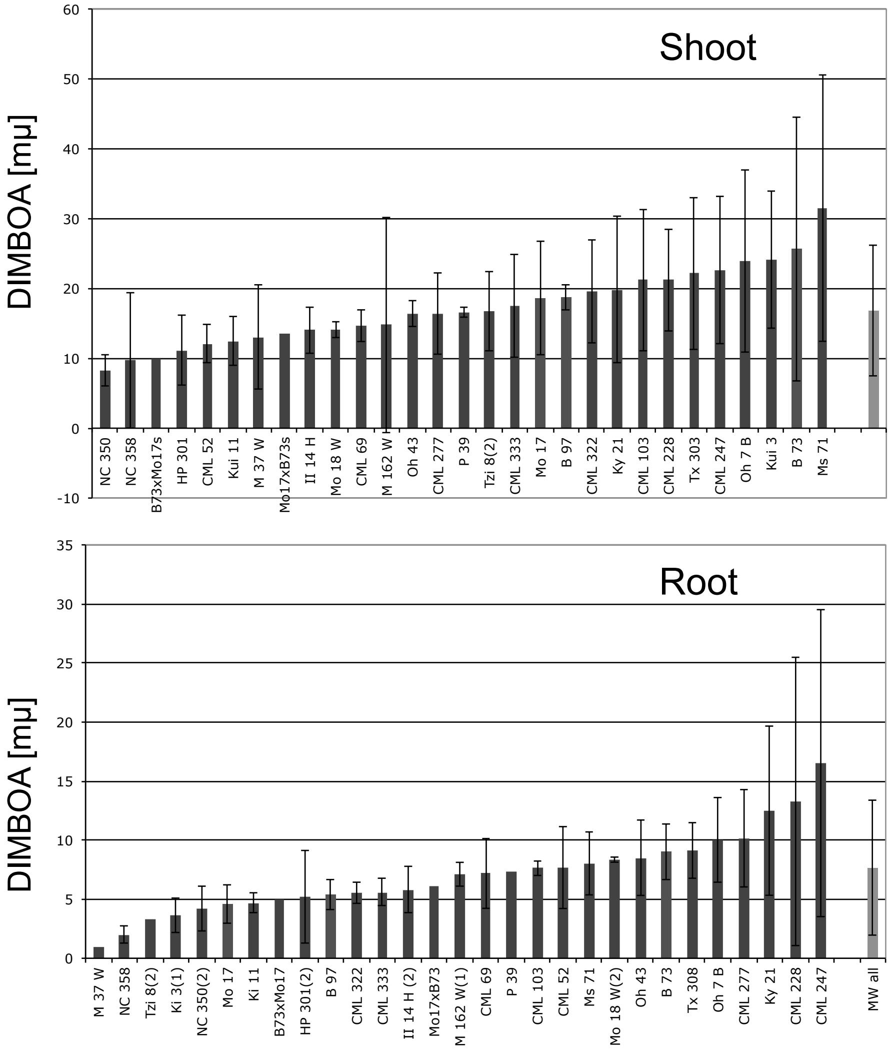

Supplemental Figure S1. DIMBOA-concentration of seedlings of the NAM panel lines. Shoot and root were analysed 4 days after imbibition (dai). The standard deviation is indicated.

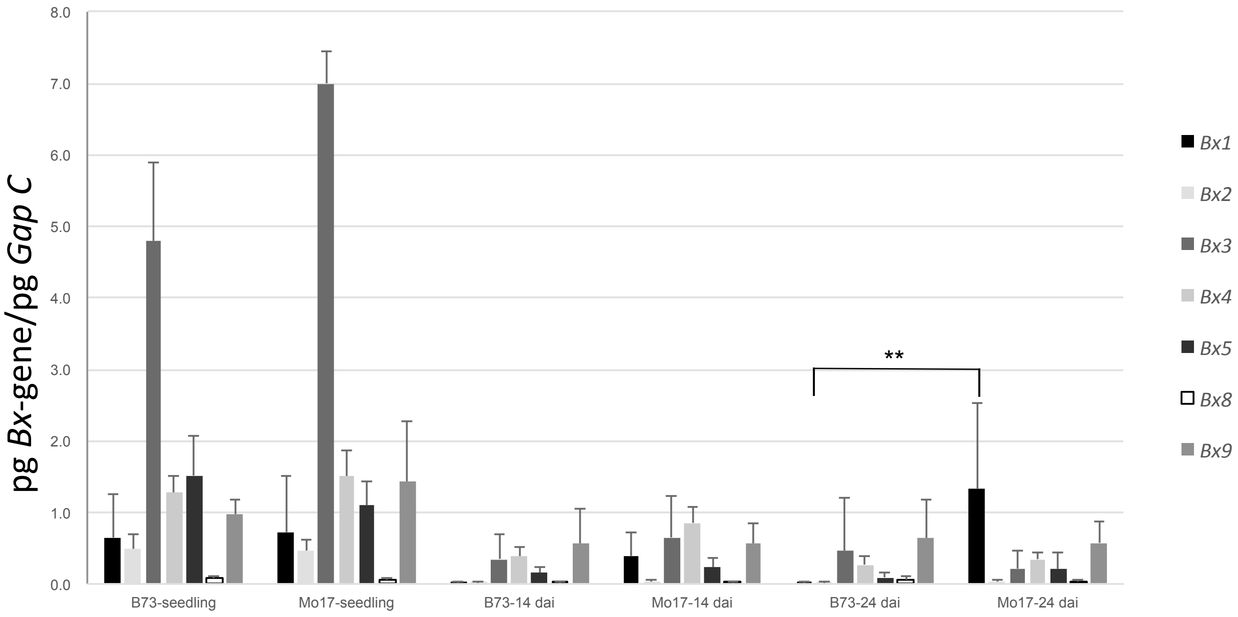

Supplemental Figure S2. Transcript levels of *Bx1* to *Bx5* and *Bx8* and *Bx9* in of B73 and Mo17. All transcript levels are normalised to *GAP C*. The standard deviation is indicated. *Bx1* transcript levels at 24 dai are significantly different with $p<0.01$ for B73 and Mo17 (Student's t-test, $n \geq 4$).

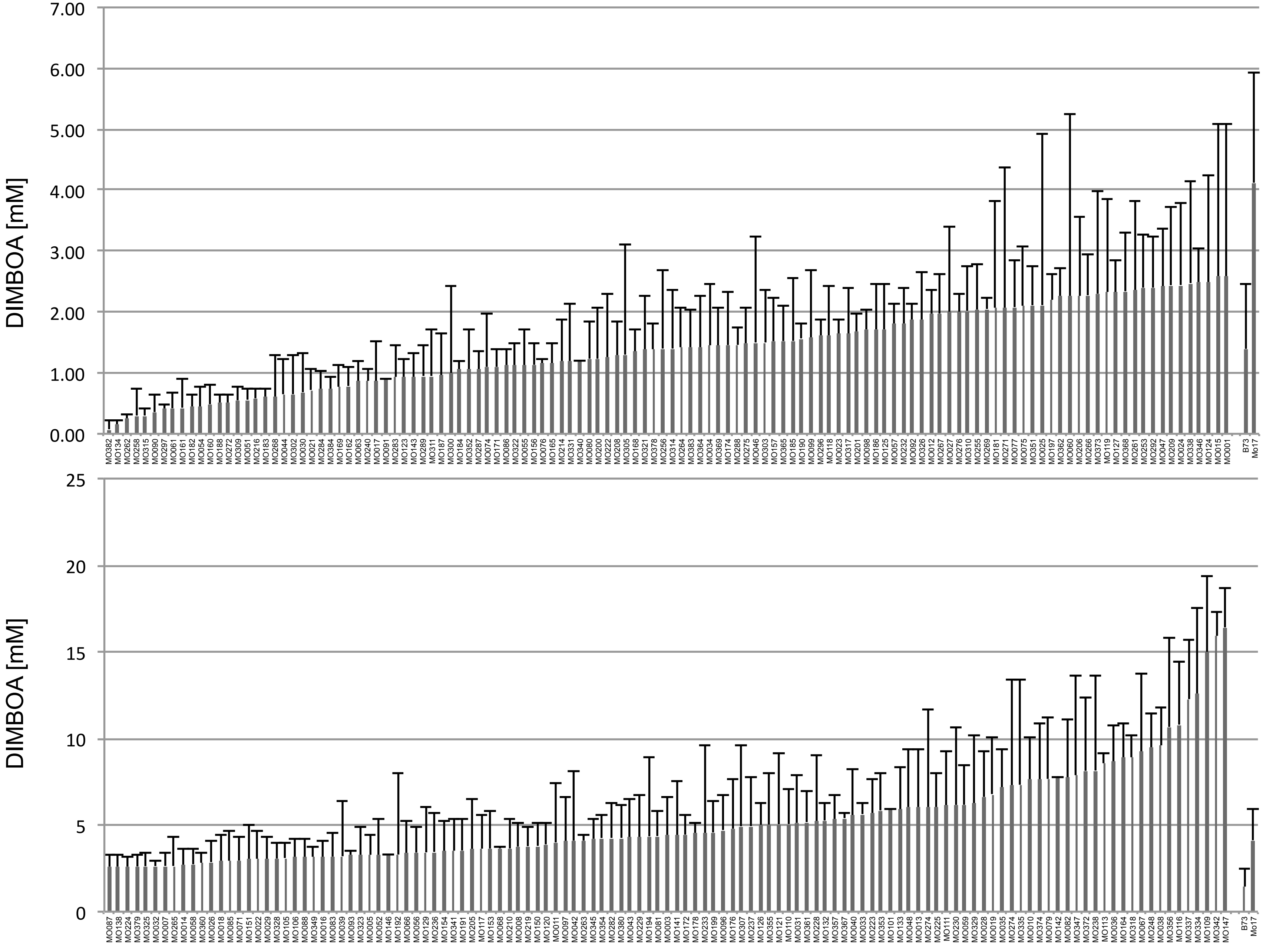

Supplemental Figure S3: DIMBOA-concentration in the IBM302 RILs, 24 dai. The standard deviation is indicated.

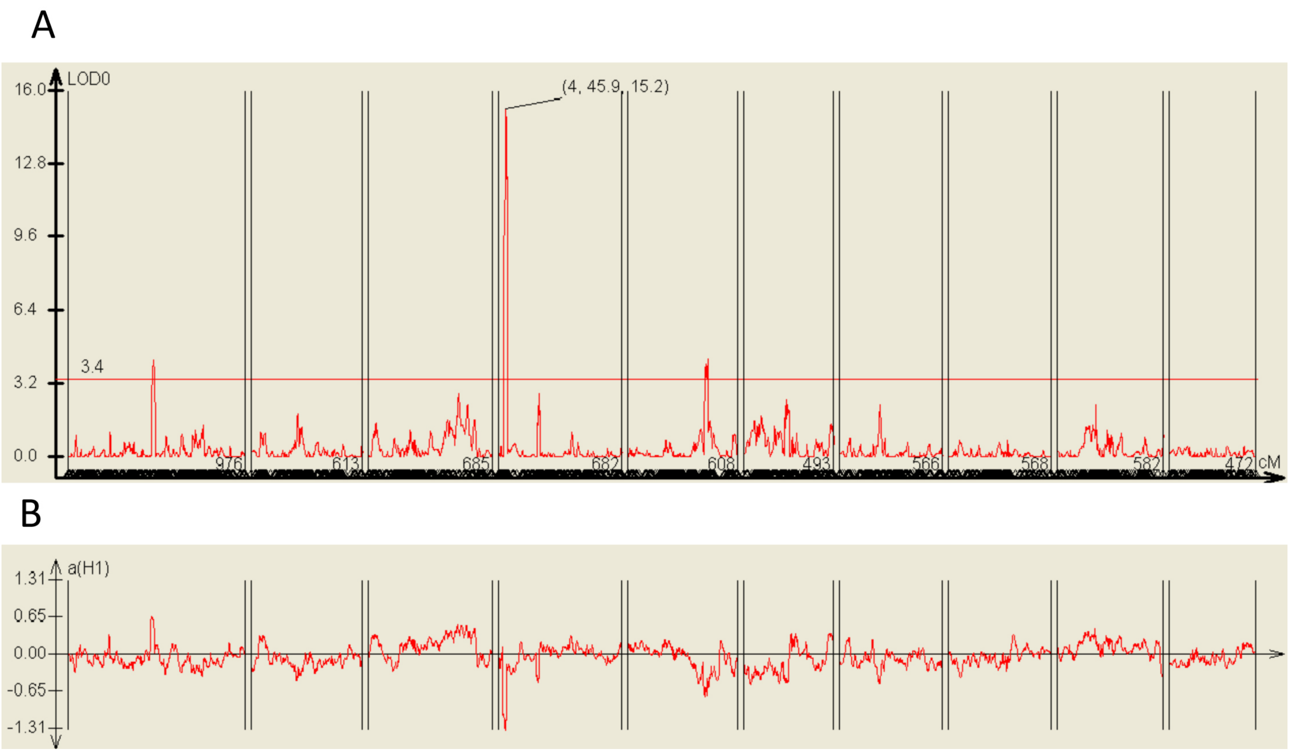

Supplemental Figure S4: QTL-analysis for DIMBOA-content in 24 dai plants with 241 IBM302 RILs. (A) LOD scores. (B) Additive effect. Values >0 contribution by B73, values <0 contribution by Mo17.

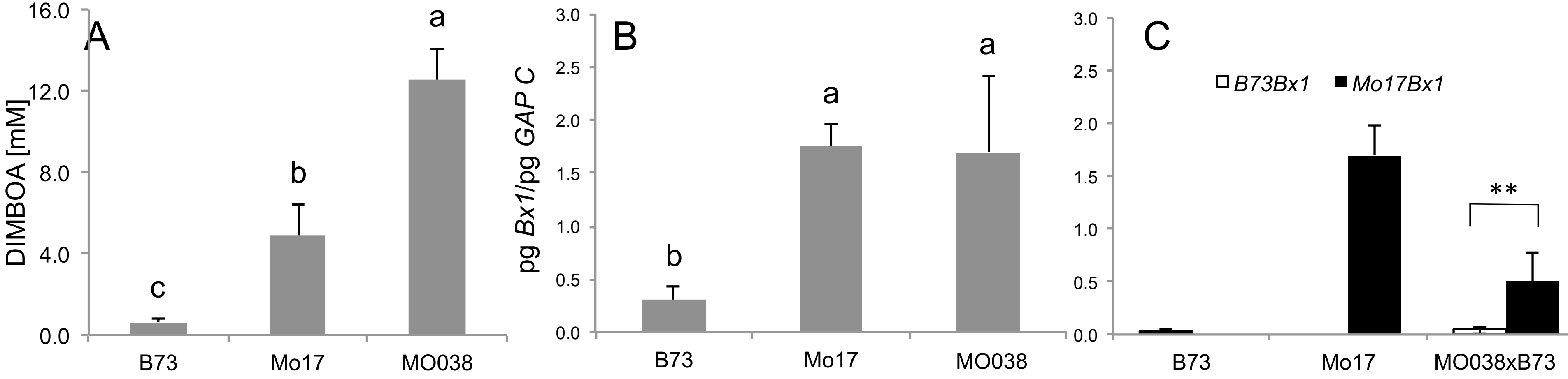

Supplemental Figure S5. Characterisation of the RIL MO038. (A) DIMBOA-content in 24 dai plants. (B) (C) transcript levels. (B) *Bx1*-transcript level of 24 dai plants. (C) Allele-specific *Bx1*-transcript level in 24 dai plants. White shading B73-allele, black shading Mo17-allele. All transcript levels are normalised to *GAP C*. The standard deviation is indicated. Data in (A) and (B) were analysed with Kruskal Wallis test and multiple comparison of treatment (n≥9 and n=4, respectively). Identical letters indicate no statistical differences (p>0.5). B73 and Mo17 *Bx1*-alleles have different expression levels (p<0.01 students test, n=4).

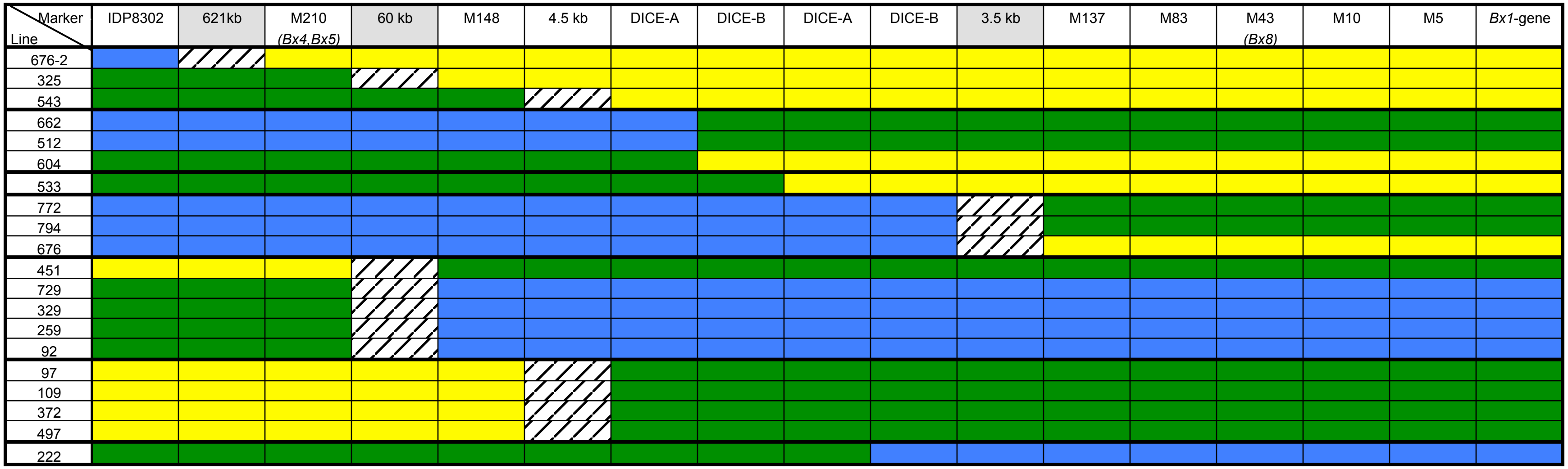

Supplemental Figure S6. Genotype of recombinants. Yellow, B73 genotype; blue, Mo17 genotype; green, hybrid genotype; striped, genotype unknown, the distance between the markers is given in the header (grey cell). The distance of the marker to *Bx1* in kb is indicated by the marker name (Supplemental Table S3) . The recombinants 676-2 and the 325 homozygous progeny were low with respect to DIMBOA-content and *Bx1*-transcript level. In the two hybrids generated by crossing with Mo17 the *Bx1* gene of the recombinant chromosome is low according to the B73 genotype. The two lines were not further analysed.

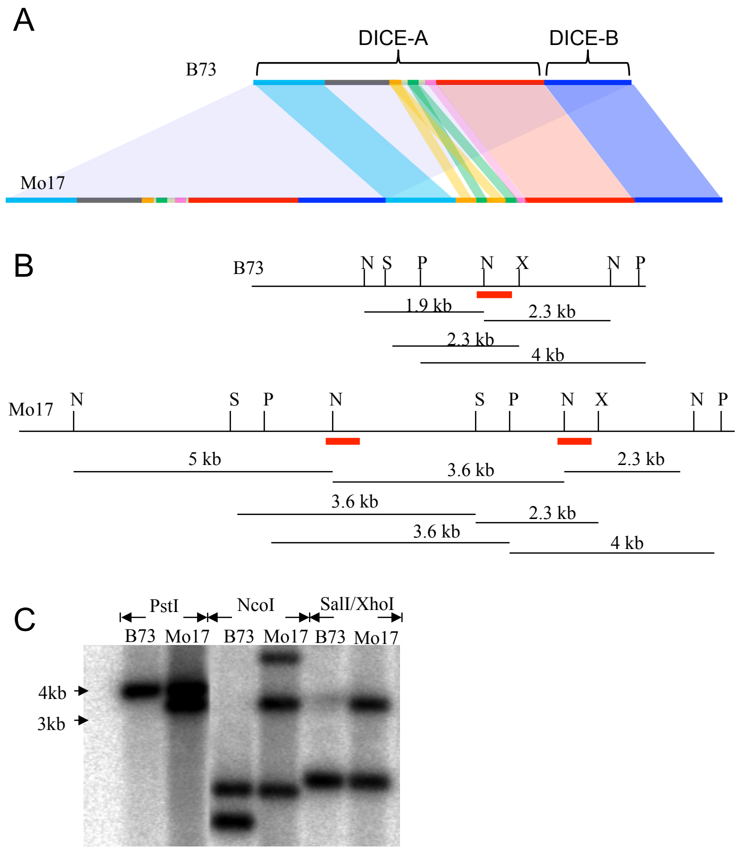

Supplemental Figure S7: Characterisation of the distal *cis*-element (DICE). (A) Schematic presentation. Formally two parts can be distinguished, consisting of 2.2 kb (DICE-A) and 1.2 kb (DICE-B, blue). Both Mo17 DICE-B sequences are identical and differ from the B73 single sequence by a 4 bp insertion, two 1bp deletions and 9 single nucleotide polymorphisms (SNPs). The first Mo17 DICE-A has a 21 bp deletion and 2 SNPs compared to B73. By contrast the second Mo17 DICE-A is greatly altered. The first 735 bp (turquois) and the last 1106 bp (red) have homologies of 84 and 94% with 5 and 4% gaps to the B73 sequence, respectively. Between these conserved regions major rearrangements took place. About 500 bp of the B73-sequence are missing (grey line), a sequence element of about 350 bp (yellow and green) present in B73 is altered (87 to 95% homology) and present in tandem duplication. DICE is a unique sequence in the maize genome. (B) Restriction map of DICE in B73 and Mo17. Restriction sites N *NcoI*, S *SalI*, P *PstI*, X *XhoI*. The red line indicates the probe used in Southern analysis. (C) Southern analysis. The restriction enzyme used and the maize lines are indicated

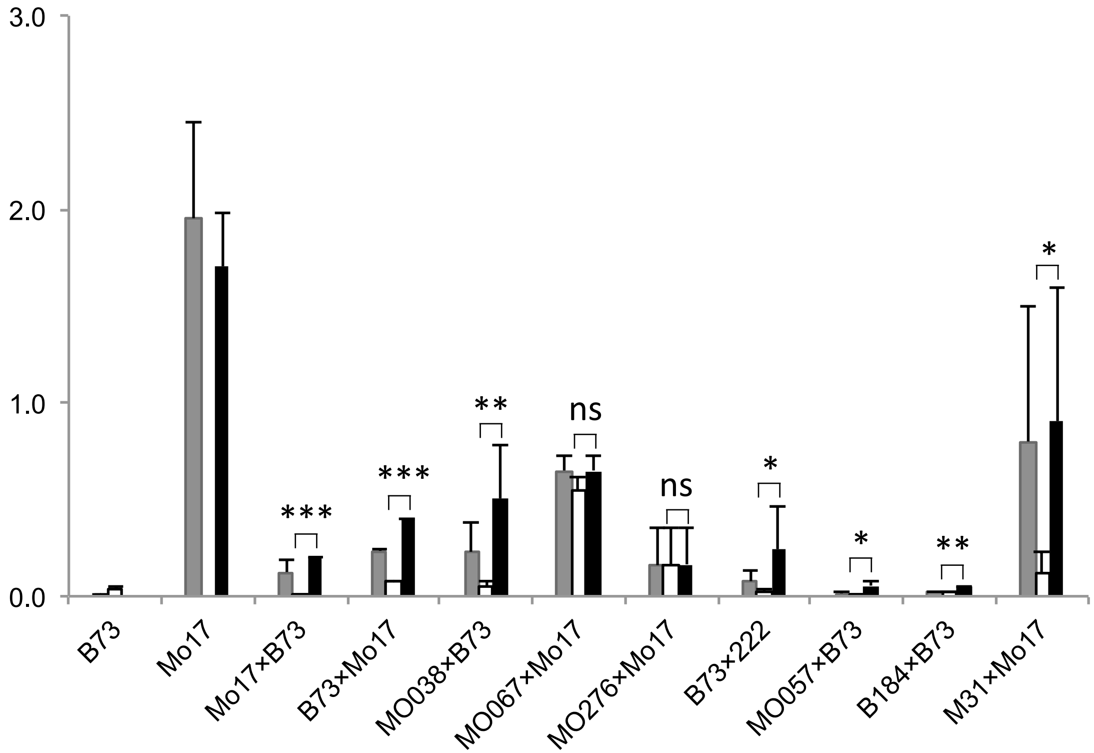

Supplemental Figure S8. Analysis of *Bx1*-transcript levels in 24 dai heterozygous plants with RIL- and NIL-parents and the recombinant 222. *Bx1*-transcript levels are normalised to *GAP C*-transcript levels. The total (grey column) and allele-specific transcript levels (white column B73, black column Mo17) were analysed in parallel. In hybrids of the RILs MO067, MO276 the Mo17 and B73 *Bx1*-alleles are equally expressed. The recombinant 222 and MO057 have identical genotype for chromosome 4. For both expression of the Mo17 allele is dominant. The *Bx1*-expression level is reduced compared to Mo17 and different for the two lines. Mean values and standard deviation are given. Significant differences in transcript levels for the B73 and M017 allele are indicated * $p<0.05$, ** $p<0.01$, ***$p<0.001$; n=4 (Student's t-test).

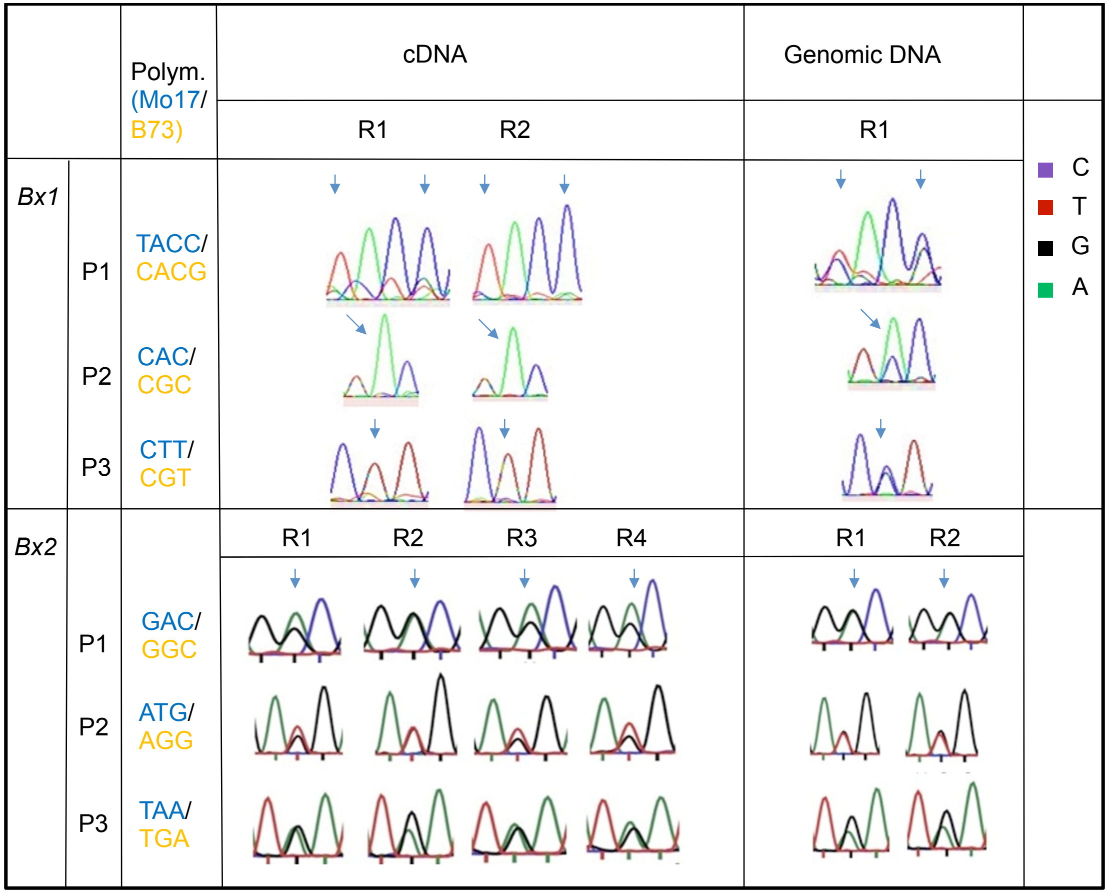

Supplemental Figure S9: Analysis of *Bx1* and *Bx2* allele-specific expression by sequencing. SNPs in three different positions (P1, P2, P3) were selected for both genes. Genomic DNA as a control and cDNA of 24 dai hybrid plants was used for amplification of gene sequences employing primers that fit for both alleles (Supplemental table S4). The amplificates were sequenced. Two to four biological replicates were analysed. The arrows point to the SNP in the sequence chromatogram.

A

B

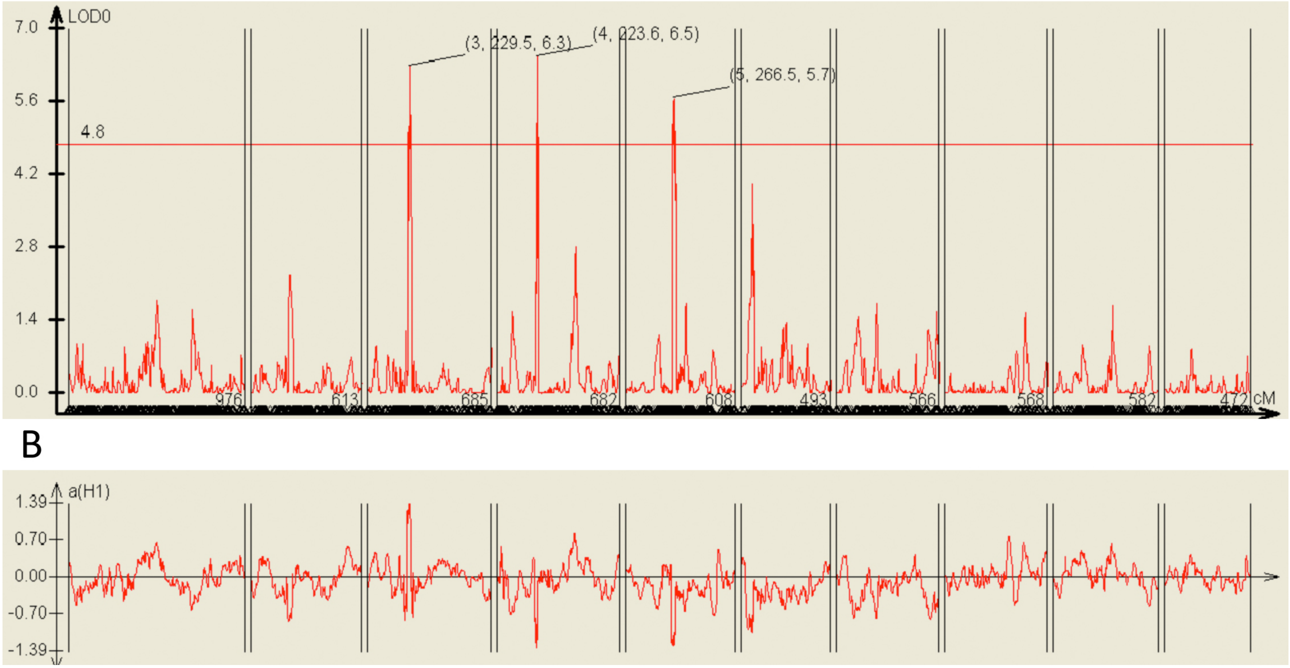

Supplemental Figure S10: QTL-analysis for DIMBOA-content in a subset of IMB302 RILs. Only RILs that have MO17-genotype for *QTL4-1* *used in the analysis*. (A) LOD scores. (B) Additive effect. Values >0 contribution by B73, values <0 contribution by Mo17.

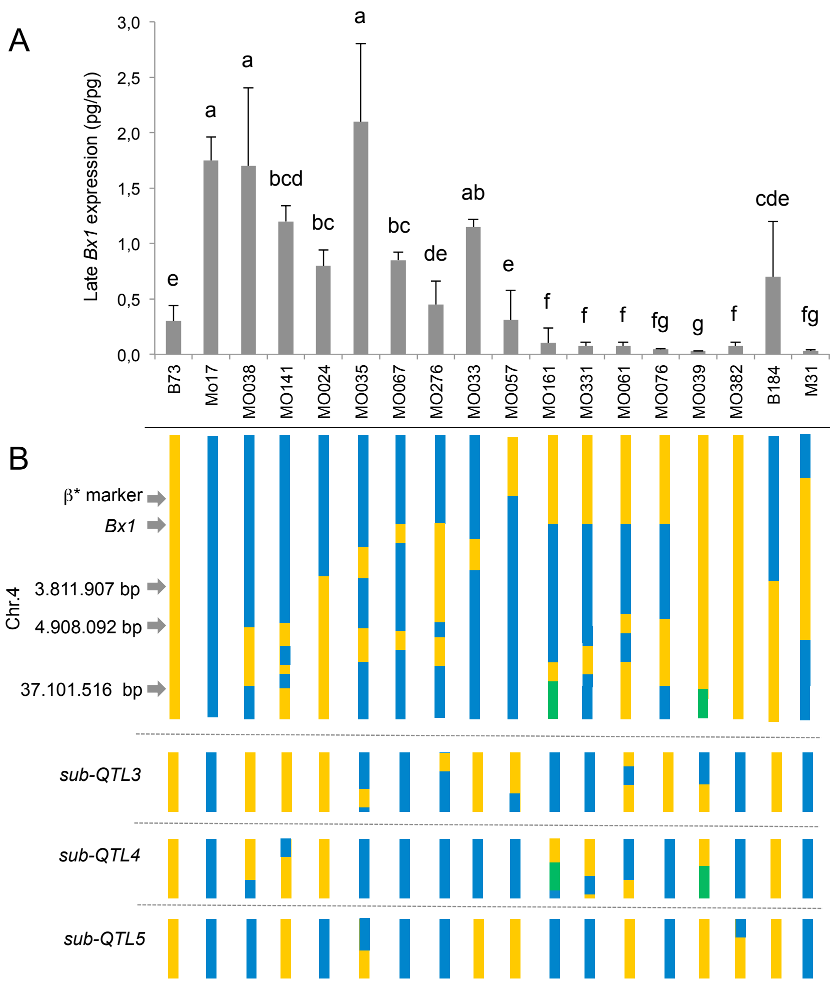

Supplemental Figure S11. *Bx1* expression analysis and genotype of selected IBM lines. (A) Relative *Bx1* expression level in 24 dai plants (see Figure 5 for details). (B) Genotype of selected IBM lines for the sQTLs determined by the IBM302 sub-population analysis using only IBM lines that have Mo17 genotype at the DICE to *Bx1*-region. Genotype: Mo17 blue, B73 yellow, hybrid green.

Supplemental Table S1. Primer and PCR-conditions

A Primers for qRT-PCR with B73, Mo17 and hybrids.

Gene	Primer	Sequences	Annealing (°C)	Extension (s)	Additive
Bx1	Bx1QF2	CGCAGCTTGGCCGAGATGAAA	50	27	5% DMSO
	Bx1QREV2	TWCTTTGTCATGGACTCATGGC			
Bx2	Bx2QF	CCGGGAGCTCACCGACATCAA	66	25	-
	Bx2QR	CTCCTGCCCCGCCGGCACGTT			
Bx3	Bx3FW4	CGGAACAGGCTGTTCAGCGAG	65	25	-
	Bx3REV4	TTCTTGCCGTCCGGCGAGC			
Bx4	Bx4QF	GCTCTCCGGCGGCAAGCAG	64	27	1M Betaine 5% DMSO
	Bx4QR	CCGTACATGTCGACCTCGGC			
Bx5	Bx5QF	TGGAGATGAGGAAGCTGTCC	64	27	1M Betaine 5% DMSO
	Bx5QR	CCGCTGTAGCTGTTGGACTT			
*Bx8**	Bx8seqFW3	TTCTTCACCCACTGCGGCTG	62	30	5% DMSO
	Bx8seqREV3	ACAAGGATCGATCAGTAGGA			
Bx9	GT1F	TCGTCACCACGCTGAACGCCAG	68	30	5% DMSO
	GT1UTAR	GGATCCTCCTTGCGCTCCTCTTTC			
GAPDH	GAPCF	GCTAGCTGCACCACAAACTGCCT	65	27	5% DMSO
	GAPCR	TAGCCCCACTCGTTGTCGTACCA			
Bx1 B73 specific**	B73BX1F:	ACATCACCGCGGGCGACCCC	72	10	5% DMSO
	B73BX1R	AGGGGTCCGAGCAGGGCA			
Bx1 Mo17 specific**	Mo17Bx1F	ACATCACCGCCGGCGACCCG	72	10	5% DMSO
	Mo17Bx1R	AGGGGTCCGAGCAGGGTACC			

* Primer pairs also used for analysis of the NAM-lines

**Primer pairs also used for fine mapping

Table S1, continued

B Primer for fine mapping.

Distance to *Bx1*/marker	Primers	Sequences	Annealing (℃)	Extension (s)	Additive
2kb	up1p2FW	GAGCAATGTCAACCTTTGGC	66	25	1M Betaine
	up1p2REV	ATGGTGGCTGCAGAAGGGAT			
3kb	up2p5KBFW	CTGCCATAGGAGCAGGGTAA	65	25	1M Betaine
	up2p5KBREV	CCCCTTCTCTCCCCTCCTTT			
5kb	MarkerA5kbB73F	CTCGTGATTCTTGTCTACTG	54	30	-
	MarkerA5kbB73R	AGGTTTGTATGGATCGACCA			
	MarkerA5kbMo17F	CTCGTGATTCCTGTCTACTA	51	30	1M Betaine
	MarkerA5kbMo17R	GTTTGTATATGGATAGACCT			
10kb	10kbB73Fw	ATGAAATGGTCAAGTTCA	58	20	1M Betaine
	10kbB73REV	GTTTGTGATTTTGTGTACAT			
	10kbMo17Fw	ATGAAATGATCAAGTTCG	58	20	1M Betaine
	10kbMo17REV	ATTTGTGATTTTGCGTACAG			
32kb	Bx8123kbFw	TCGAGAGGGACGGACTTAAC	68	30	1M Betaine
	21kbdownbx8REV2	TGCCAATCATGGCGGTTTCA			
43kb	Bx8up3F2	AGCCCTAGATCGCCAGGGA	68	30	1M Betaine
	Bx8up3R2	CTAGCACCTTGGGTGCGCT			
83kb	up80kbBACRev	TGGCAAGTCAAGAACAGAACC	65	27	1M Betaine
	up80kbBACFW	GGAGAGGAGGATGCTGCTTA			
137kb	3119224F3	TGTTTGGCTAGCTGTCGGAT	57	50	1M Betaine
	3119224R3	GGAGGGAGTACCTTGTGTAT			
DICE-marker	DICEFW	ACCATGCATGCTGAGAGAGA	57	50	1M Betaine
	DICE2REV	AGTTGCACCGGAGCACATAT			
143kb	3113F14F2	GAGGAGCTCGACCGGTCAG	57	20	1M Betaine
	3113F14R2	TTTGTAGGGTTTGGGTGGGT			
148kb	Marker2B73F	AGGGATCTATGCTTGGGGAA	57	30	1M Betaine
	Marker2B73R	GAACGGATATTCATAATATT			
	Marker2Mo17F	AGGGATCCATACATGGGCAG	57	30	1M Betaine
	Marker2Mo17R	ACGGATATTCATAGTGGACA			
210kb	MarkerBM1B73F	CAAAAAATCTAGAATCTCAA	50	40	-
	MarkerBM1B73R	GGGATGACTAAGTCATGTCA			
	MarkerBM1Mo17F	TCAAACATCTAGAATCCCAT	50	40	-
	MarkerBM1Mo17R	GGGATGACTAAGCAATGTTG			

Table S1, continued

C Primer for the analysis of NAM lines.

Gene	Primer	Sequences	Annealing (°C)	Extension (s)	Additive
Bx1	Bx1QgenFW	ATGGCTTTCGCGCCCAAAACG	60	16	5% DMSO
	Bx1QgenREV	GGCTCCTCCTCTCGGCGGGT			
Bx4	Bx4QgenlFW	CCGGGAGCTCACCGACATCAA	62	16	5% DMSO
	Bx4QgenREV	GTGGCCGTACTTGGCGTGGA			

D Primers for determination of the genomic sequence of the DICE region in Mo17.

Primer	Sequences	Annealing °C	Extension (s)	Additive
MetFW3	CACAGTATTATTTGCTCCGT	56	100	1M Betaine
ChipR1	GACAGGGTTGTTGTATATGCA			
Mo17D1D2ChipF1	GGGCGCCATTGGCATTGTTG	50	50	1M Betaine
gMo17R1	GTCGTGTTCACACTCAACAT			
151AMo17F1	GGTGCATGTGCGAGAAAACC	56	120	1M Betaine
3116KF9	GCACACTCTCAACACTACAA			
gMo17F2	GTTAGAACTTGGTAGCGAGT	52	50	1M Betaine
MetREV3	TGCCATCGATATCAAGGCAG			
151AB73F1	GGTGCATGTGCGACAACCTG	52	120	1M Betaine
gMo17R2	CAGTGACATATGTTCTCAGT			
gMo17F1	CCACATATTTCCAAAATCGA	52	120	1M Betaine
MREV3	TGCCATCGATATCAAGGCAG			
gMo17F4	GTTGAGTGTGAACACGACAT	53	90	1M Betaine
MREV3	TGCCATCGATATCAAGGCAG			
gMo17F4	GTTGAGTGTGAACACGACAT	53	90	1M Betaine
MREV4	TCACCCCATCCTAAATAAGG			

Supplemental Table S2. Composite Interval Mapping

		QTL cartographer data					
QTL	Chromosome	Marker interval[a]	Confidence interval	Peak position	LOD	Add. effect	R2(%)[b]
QTL1	1	lsu146-bnlg2057	176.512.769-185.017.139	umc1972	4.2	0.67	5.6
QTL4-1	4	SYN17726-SYN1073	2.765.157 - 3.452.186	PUT-163a-94477564-4893	15.2	-1.27	21.5
QTL4-2	4	npi386-PZE-104031917	2.710.598-38.725.625	PZE-104029222	2.7	-0.49	3.3
QTL5	5	umc126a-npi458a	90.689.469 - 200.494.124	php20531	4.3	-0.7	6.1

The threshold calculated by 1000 permutation is 3.4.

a Marker pair bordering the QTL
b Phenotypic variation explained by each individual QTL

Supplemental Table S3. MO038 Genotype

QTL	Confidence interval (bp) *	Positive contribution	Genotype MO038
QTL4-1	3,046,940 to 3,257,533	Mo17	Mo17
QTL4-2	22,719,598 to 38,725,625	Mo17	B73: 22,719,598 to 37,101,516<br>Mo17: 37,101,517 to38,725,625
QTL1	176,512,769 to 185,017,139	B73	B73
QTL5	190,689,469 to 200,494,124	Mo17	Mo17

* based on AGI's B73 RefGen_v2

Supplemental Table S4. Marker for fine-mapping

Name	Position (bp)	Detection	Type
Bx1-gene	3.256.736-3.256.843	in gel	plus/minus
M2	3.254.405-3.254.897	sequencing	SNP
M3	3.253.475-3.253.979	sequencing	SNP
M5	3.250.998-3.251.489	in gel	plus/minus
M10	3.246.208-3.246.410	in gel	plus/minus
M32	3.224.206	sequencing	SNP
M43	3.214.107-3.214.245	sequencing	indel
M83	3.173.538-3.173.540	sequencing	indel
M137	3.119.173-3.119.728	sequencing	indel
DICE	3.115.056-3.115.570	sequencing	duplication
M143	3.113.527-3.113.362	in gel	indel
M148	3.108.448-3.108.129	in gel	plus/minus
M210	3.045.994-3.046.270	in gel	plus/minus

Supplemental Table S5. *Bx*-gene transcript levels in transgenics at 24 dai

Genotype	Transcript levels (pg/pg *GAP C*)					
	Bx1	*Bx2*	*Bx3*	*Bx4*	*Bx5*	*Bx8*
WT-1	0.5±0.1	1,76±1,16	4,02±2,07	2,08±0,88	0,32±0,14	1,34±0,34
OE-1	26.1±14.4	10,5±14,87	6,56±4,10	2,30±1,18	0,69±0,75	1,89±1,86
WT-2	0.2±0.5	3,52±3,73	4,80±2,53	1,16±0,51	0,42±0,21	1,40±0,33
OE-2	24.4±17	1,26±1,11	2,98±1,59	0,67±0,22	0,23±0,06	1,10±0,40

List of markers

Marker ID and position on the chromosome in cM are given.

C1; 230 marker
umc1354 0.0000 tub1 2.5000 umc1177 10.5000 umc1566 16.5000 bnl5.62a 22.7000 mmp102 25.9000 umc94a 33.1000 lim179 42.4000 mmp49 51.4000 cdo1081b 53.1000 asg31 60.5000 npi415 61.3000 php20537b 64.7000 ufg31 66.9000 ufg33 67.3000 ufg32 68.1000 ufg34 69.7000 bnlg1014 76.4000 umc1363 77.3000 umc1071 78.7000 umc1269 79.7000 umc1977 82.6000 php20603 85.1000 php20689 99.8000 umc1685 101.7000 mmp93 101.9000 umc1160 106.9000 npi579b 110.1000 umc157 112.7000 bm3 113.6000 mmp68 121.7000 csu1171 125.5000 umc1166 131.8000 umc1568 140.0000 bnlg1429 141.7000 php20640 145.1000 lim504 149.8000 umc1976 158.8000 mmp135 164.2000 bnlg1127 165.7000 bnlg1953 168.2000 npi403b 182.0000 umc76 196.6000 cdo1387b 199.7000 lim122 203.2000 umc1073 206.7000 umc1403 208.8000 npi439a 215.0000 umc230 216.6000 umc11a 217.0000 mmp66 222.6000 umc1397 224.6000 PZE-103055522 229.0000 npi242b 233.9000 umc1479 236.2000 bnlg1203 238.2000 umc1021 239.5000 umc13 242.2000 asg35b 246.0000 ndp2 254.7000 mmp133 258.3000 mmp23 258.3000 ndp1 260.7000 bnlg1866 262.2000 umc1598 262.8000 lim432 264.6000 mmp151a 269.4000 mmp100 283.6000 mmp56 293.1000 cdo938a 295.3000 bnlg2238 298.9000 umc1849 307.2000 asg75 308.7000 umc1169 309.7000 asg30b 312.7000 csu207 321.5000 ufg13b 331.2000 ufg77 335.0000 isu041b 338.9000 isu061b 340.9000 ufg43 345.8000 bnlg1811 348.6000 bnl9.11b 353.0000 bnlg2295 360.4000 asg3 364.1000 csu3 367.2000 mmp61 374.8000 lim497 375.9000 umc2025 379.2000 mmp39 387.4000 umc1515 393.0000 uaz246c 397.7000 mmp143 399.4000 csu1138 400.6000 umc1076 401.7000 mmp101 402.8000 umc1676 404.9000 umc1703 407.8000 umc1124 410.9000 cdo344c 414.5000 umc1906 420.8000 mmp124 423.1000 umc1395 424.3000 umc1321 426.1000 umc1601 426.7000 umc1603 428.3000 hac101b 434.1000 uaz276 438.1000 umc67a 448.8000 isu146 453.4000 umc1972 455.5000 myb6 466.9000 umc1590 469.2000 bnl5.59 472.6000 php20654 475.4000 asg58 477.5000 umc1811 478.3000 bnlg2057 479.4000 bnlg1598 484.6000 umc1123 487.3000 mmp156 495.7000 umc1396 500.5000 umc1748 505.2000 umc1919 507.6000 bnlg1615 509.4000 psr152a 511.6000 umc2151 515.7000 ntf1 522.6000 umc58 527.7000 asg16b 530.0000 mmp123 535.1000 php20644 541.5000 umc1924 545.7000 ufg50 549.4000 uaz147b 551.8000 csu1132 555.5000 umc1925 557.7000 asg62 558.5000 csu374b 573.7000 php20855 578.6000 bcd98a 580.8000 umc1358 584.7000 bnlg1556 588.8000 umc23a 600.3000 lim442 615.2000 mmp189 620.5000 mmp173 623.6000 php20661 629.9000 bnlg1025 630.5000 bcd207a 633.9000 umc1128 637.3000 umc1147 640.2000 bcd386a 643.6000 npi447a 646.1000 phi002 646.7000 umc1245 647.7000 umc128 648.2000 umc37a 651.9000 cdo98b 659.6000 mmp99 664.7000 bnlg2228 669.9000 umc83a 671.2000 lim254 675.0000 npi120 680.6000 umc1955 685.1000 umc2181 689.2000 npi255 689.9000 umc1838 692.1000 csh4 693.9000 umc1446 696.3000 umc1928 697.9000 an1 699.7000 umc1991 710.2000 umc1383 714.8000 cdj2 721.7000 ufg53 724.6000 csu696 730.9000 rz403 735.2000 chrom7 742.4000 phi011 748.7000 umc2047 751.7000 umc140a 755.3000 bnlg1331 756.3000 csu222a 756.6000 msu2 762.3000 umc197a 764.8000 csu554a 774.4000 umc1082 782.7000 umc1431 786.1000 umc107a 786.9000 cdo122a 787.6000 nfa103a 792.0000 mmp141 793.5000 umc2149 798.4000 mmp83 798.9000 bnlg1671 802.9000 mmp172 804.7000 npi407 806.2000 rz630a 809.6000 lim99a 815.8000 npi282b 817.4000 lim78 820.0000 uaz130a 824.0000 mmp87 831.9000 lim39 839.7000 umc161a 853.1000 phi265454 862.8000 mmp195g 871.0000 npi238 876.2000 cdo87b 877.0000 umc1553 877.8000 umc1421 880.4000 umc1681 885.1000 umc1129 889.3000 bnl8.29a 891.6000 umc1111 892.8000 npi241a 896.4000 umc1118 902.2000 umc1744 913.1000 umc84a 916.2000 umc86a 916.7000 umc1630 917.9000 lim228 947.2000 umc1331 951.3000 csu1089 952.8000 umc2045 954.1000 phi064 957.8000 bnl6.32 968.0000 umc1605 971.9000 umc2100 975.9000

C2; 128 marker
isu053a 0.0000 isu144a 24.0000 php20568b 29.4000 psb485b 38.6000 umc1165 42.8000 umc53a 46.3000 npi254a 51.0000 umc1542 53.0000 bnlg1297 55.3000 bnlg1017 61.1000 umc1980 71.0000 umc1265 73.1000 npi208c 80.8000 npi421a 80.8000 umc1824 82.8000 mmc0111 83.5000 eks1 110.2000 bnlg2277 130.9000 bnlg1327 135.9000 myb5 137.0000 umc1262 140.9000 umc1261 142.4000 umc1422 144.4000 umc6 151.1000 lim328 157.9000 mmc0231 165.6000 umc44b 168.5000 npi287a 177.7000 umc61 179.5000 umc1776 183.4000 mmp33 189.3000 psr901 198.4000 umc1185 202.7000 mmp42 204.7000 bnlg1064 209.3000 umc34 214.2000 bnlg381 215.5000 npi607 217.9000 umc1024 220.8000 umc1326 230.0000 umc259b 231.4000 umc1448 232.5000 umc1465 236.6000 umc1541 237.8000 mmp167 244.2000 prp2 247.6000 umc134b 251.4000 umc1580 253.6000 bnlg1018 253.6000 bnlg1175 254.5000 mmp91 261.9000 bnlg108 265.6000 umc1259 269.5000 umc2030 272.8000 umc1861 273.7000 npi242a 274.3000 umc2088 276.0000 bnlg121 278.6000 umc2079 280.0000 php10012 280.3000 mmp89 291.5000 umc2007 294.3000 umc1454 299.4000 umc131 302.5000 isu089 303.1000 psr666 304.2000 umc1003 305.0000 mmp119 306.3000 csu1080b 311.8000 mmc0401 315.9000 umc1028 318.5000 npi297 324.5000 umc1079 326.6000 bnlg1036 330.8000 umc1658 332.7000 umc2178 334.8000 bnlg1138 336.5000 umc1080 337.2000 psb365b 337.6000 umc1004 338.9000 umc1108 358.6000 bcd926b 366.4000 php20005 368.5000 umc2129 371.2000 umc1890 379.4000 rz474c 396.4000 uaz194a 399.8000 phi251315 403.4000 umc36b 409.4000 php20017a 411.4000 umc1560 412.1000 asg20 415.8000 umc1049 417.8000 mmp116 419.3000 mmp84 434.5000 umc137a 442.4000 phi435417 445.8000 umc1947 447.9000 umc1604 449.1000 bnl6.20 454.9000 npi210 461.0000 umc2085 466.4000 npi298 470.7000 mmp34 478.3000 npi610 483.2000 mmc0381 488.2000 mmp188 489.1000 bnlg1746 489.4000 isu091 491.2000 bnlg1940 493.4000 psr144a 495.3000 psr144c 496.9000 umc1516 500.1000 umc49 507.3000 umc1256 516.5000 umc1252 516.7000 mmp195e 552.6000 bnlg469b 565.9000 bnlg1893 570.6000 umc36a 576.9000 umc2184 588.5000 mmp183 590.8000 bnl17.14 598.7000 lim104 602.7000 nfa103b 604.3000 ufg55 607.2000 phi101049 608.3000 umc1696 612.5000

C3; 181 marker
umc2118 0.0000 umc2105 2.0000 umc1931 5.6000 phi453121 7.3000 phi404206 11.2000 umc1780 11.4000 bnl8.15 11.6000 umc1394 22.0000 umc1970 29.0000 umc2071 29.9000 asg64 30.3000 mmp158a 32.5000 umc1892 36.2000 php20905 37.9000 phi104127 38.7000 umc2049 39.4000 mmp38 43.3000 asg30c 58.7000 csu32 60.7000 umc1458 67.9000 bnlg1144 77.7000 umc1886 79.2000 umc1057 97.0000 php20042a 100.6000 bnlg1647 102.6000 csu324b 107.2000 asg24 108.3000 lim66 124.1000 bnlg1447 128.0000 mmp79 138.1000 mmp186 144.1000 asg48 151.5000 bnl8.35a 152.2000 npi276a 152.9000 umc1012 157.9000 umc1030 157.9000 npi446 162.7000 umc1425 163.9000 umc2000 165.8000 umc1608 166.9000 umc2158 175.6000 umc1495 176.4000 umc1392 180.1000 umc2033 180.7000 psr754b 185.0000 umc1742 188.0000 umc2117 189.2000 bnlg1452 189.8000 bnlg1113 189.8000 bnlg1638 192.1000 mmp144 195.9000 mmp36 202.5000 mmc0132 207.8000 cdo244d 207.8000 mmc0312 209.7000 umc1908 210.6000 npi247 210.8000 mmp69 212.3000 SYN20676 213.0000 SYN18257 214.0000 rz382a 214.9000 SYN33627 215.0000 SYN13461 216.0000 SYN13453 217.0000 bnlg1816 217.3000 PZE-103046325 218.0000 SYN737 219.0000 rz244b 219.9000 SYN937 220.0000 PZE-103048201 221.0000 php10016c 221.3000 PZE-103047928 222.0000 PZE-103049948 223.0000 umc1223 223.9000 PZE-103051375 224.0000 PZE-103051881 225.0000 PZE-103051979 226.0000 PZE-103053111 227.0000 PZE-103055323 228.0000 umc1920 229.4000 PZE-103056783 230.0000 umc10a 230.4000 PZE-107022749 231.0000 php20558a 231.2000 PZE-106021829 232.0000 mmp9 232.4000 umc1449 238.7000 hac101a 245.9000 umc1527 248.6000 umc1773 249.7000 psr628 249.9000 psb527a 253.2000 umc2002 260.0000 cdo344a 263.9000 phi053 265.5000 umc102 267.3000 umc1174 268.6000 umc1600 270.4000

mmp80 273.1000 uaz288a 274.7000 umc1158 276.4000 rz296b 278.9000 umc1102 282.1000 bnlg1035 282.7000 mmc0022 285.0000 umc2020 285.0000 umc1167 285.9000 umc1501 292.1000 psr119a 294.7000 cdo105 297.1000 umc26a 304.6000 csu184 307.2000 csu636 321.2000 umc1973 331.7000 npi296 337.4000 umc1539 338.2000 bnl5.37b 338.7000 umc1311 342.0000 umc1730 345.5000 umc1027 348.3000 bnl10.24a 348.9000 lim486 352.4000 rz538b 358.3000 asg39 363.4000 psb79 381.8000 umc60 382.8000 psr754a 389.6000 umc1644 403.5000 mmp5 407.9000 csu1183 410.4000 bnlg1951 411.9000 umc82c 412.6000 ufg42 416.5000 csu191 418.3000 bnlg1160 421.7000 lim424 432.7000 php15033 438.9000 bnlg197 439.4000 asg7b 443.4000 bnl6.16a 447.1000 umc2050 464.4000 umc1135 466.4000 umc1767 468.2000 umc1528 470.6000 bnlg1605 470.8000 umc1399 470.8000 npi212b 471.2000 umc1489 484.7000 umc1404 485.4000 php20521 489.8000 umc1825 496.6000 umc17a 502.5000 umc1140 526.3000 mmc0251 528.0000 umc1915 533.7000 bnlg1108 534.8000 umc2081 543.3000 php10080 548.6000 umc1320 550.0000 umc1273 551.0000 umc2174 566.2000 umc63a 573.3000 csu303 575.3000 csu845 580.0000 psb107c 606.4000 psb443 610.8000 psb041 610.8000 sho89 611.0000 umc2152 616.9000 lim182 621.2000 umc1813 626.7000 bnlg1536 630.2000 bnlg1754 635.0000 npi457 636.3000 npi425a 638.3000 npi420 647.2000 umc1641 663.1000 lim444 673.3000 lim96 679.9000 lim82 681.1000 umc1594 685.1000

C4; 157 marker
ufg26 6.7000 bnlg1434 9.0000 ufg52 11.7000 rca1 14.6000 msf1 20.5000 csu221 23.4000 SYN22929 26.0000 SYN17726 27.0000 SYN17710 28.0000 umc1228 28.1000 umc123 29.3000 SYN9499 31.0000 mmp174 35.1000 SYNGENTA0009 37.4000 PUT-163a-94477564-4893 38.1000 bx4 42.2000 PUT-163a-148946720-494 46.9000 SYN19236 48.1000 SYN1073 50.0000 SYN1064 50.3000 umc1164 52.3000 PZE-104004499 54.1000 umc1669 62.2000 phi295450 85.7000 php20725 88.4000 umc1943 105.8000 psr144b 134.8000 umc87a 138.8000 umc1926 145.6000 csu235 148.2000 isu144b 160.6000 umc2176 168.1000 umc1902 174.4000 mmp111 182.1000 umc2039 189.4000 pgd3 193.3000 wip2 198.0000 npi386 203.7000 umc1117 211.9000 lim415 217.0000 umc1963 219.2000 PZE-104028825 221.0000 umc1652 221.9000 PZE-104029222 222.0000 SYN1245 223.0000 psb527b 223.9000 PZE-104029568 224.0000 SYN14310 225.0000 mmc0471 225.7000 SYN14309 226.0000 SYN24546 227.0000 PZE-104030661 228.0000 SYN24109 229.0000 SYN24105 230.0000 PZE-104031501 231.0000 bnlg490 231.3000 PZE-104031917 232.0000 SYN8860 233.0000 SYN4890 234.0000 SYN25580 235.0000 PZE-104033459 236.0000 agrr301 239.0000 umc1969 242.1000 umc2061 244.3000 phi026 249.1000 phi079 249.1000 umc191 253.5000 bnlg1265 263.3000 umc1303 265.1000 psr152b 268.1000 csu509 270.2000 mmp125 273.6000 umc1031 276.3000 umc42a 278.7000 isu061d 281.6000 bnl15.45 283.2000 umc1511 285.5000 psr128 287.7000 mmp140 289.2000 bnlg1755 290.2000 umc1142 292.9000 umc1346 294.7000 mmp149 296.8000 mmp78 301.3000 php20597a 309.0000 mmc0371 310.5000 umc1945 312.2000 umc2027 328.9000 mmp97 333.5000 mmp176 334.4000 zm1 340.4000 rz567b 346.7000 rz273a 352.6000 bnlg1137 365.6000 umc66 370.6000 umc104a 381.9000 mmp147 384.0000 umc2038 384.5000 umc19 387.4000 mmp115 393.8000 bnl5.24b 403.4000 asg33 410.7000 umc1775 416.4000 umc1667 422.6000 umc1808 426.1000 mmp3 429.1000 asg27a 431.3000 bnlg1444 435.7000 gol1 436.5000 bnl10.05 439.3000 bnlg2244 440.3000 umc1899 443.9000 umc158 447.2000 npi570 453.8000 ufg23 474.8000 npi270 483.0000 php20071 484.3000 phi093 488.3000 npi444 490.3000 umc15a 492.0000 umc2187 497.9000 umc1842 500.8000 rz596b 503.1000 umc2135 508.3000 mmp178 515.2000 umc2188 518.3000 umc52 523.2000 lim446 529.6000 umc2139 539.0000 php10025 544.0000 umc1999 546.0000 umc1854 559.9000 mmp134 563.8000 npi449b 565.9000 asg22 567.6000 mmp94 569.8000 umc1328 574.3000 cdo534a 577.6000 umc1573 594.1000 npi593a 601.2000 umc1101 604.4000 umc2046 606.4000 php20608a 619.2000 bnlg589 619.6000 umc1532 621.3000 umc124b 621.8000 umc1109 637.2000 lim471 638.1000 bnl15.07a 640.6000 asg41 644.9000 umc1180 648.3000 umc169 660.3000 umc1058

669.1000 mmp182 672.0000 umc1197 675.4000 umc1707 679.7000 bnlg1890 681.6000

C5; 153 marker
ufg36 0.0000 umc1253 1.9000 csu1087 3.0000 umc1423 8.5000 umc1445 16.1000 umc1097 19.3000 mmp6 20.9000 bnl8.33 25.3000 umc1901 28.6000 umc86b 31.3000 umc1325 33.1000 umc1260 46.6000 sca1 47.0000 npi409 48.0000 umc1523 50.4000 bnl7.21c 52.9000 psb239a 62.0000 lim407 63.3000 umc2036 96.7000 npi282a 101.1000 rz630f 109.8000 tua4 119.3000 asg73 121.5000 bnlg565 122.7000 psr922a 125.6000 umc1587 128.7000 umc107b 132.0000 cdo122b 136.4000 mmp130 151.4000 bcd1072a 153.5000 bnlg1879 161.8000 csu164b 176.4000 rz474a 177.1000 umc1686 183.4000 mmp112 185.8000 cdo795b 187.6000 bnlg1046 189.4000 umc1597 190.9000 bnl7.56 192.3000 csu340 195.6000 psr544 200.3000 bcd207b 202.1000 mmc0351 203.4000 umc2035 204.0000 cdo98a 207.8000 bnl5.02a 209.3000 umc1048 210.6000 umc1447 215.6000 lim175 221.7000 umc1315 227.6000 ufg49 232.7000 bnl6.10 233.9000 umc43 233.9000 umc1935 235.3000 rz242b 238.1000 umc1609 243.5000 umc1 246.4000 umc1355 246.9000 PZE-105063794 249.0000 mmp108a 250.0000 PZE-105064348 251.0000 umc1389 252.2000 psr167 252.4000 PZE-105065040 253.0000 PZE-105065758 255.0000 php15024 255.0000 ufg60 256.9000 SYN38052 257.0000 SYN29079 259.0000 mmp58 260.7000 PZE-105069355 261.0000 SYN26103 263.0000 bnlg1902 263.2000 PZE-105071013 265.0000 SYN9330 266.0000 PZE-105072411 267.0000 bnl4.36 267.3000 PZE-105073571 268.0000 PZE-105074598 269.0000 SYN8956 271.0000 PZE-105076945 272.0000 PZE-105077796 272.5000 umc40 274.2000 mmp60 276.4000 rz87 279.1000 umc1591 280.4000 umc1060 283.1000 umc1990 284.5000 umc1747 286.6000 bnlg1208 288.7000 lim4 289.9000 SYN27038 292.0000 PZE-105107801 294.0000 bnlg2323 294.1000 PZE-105107897 296.0000 npi449a 297.0000 SYN37477 298.0000 PZE-105108343 300.0000 SYN38246 302.0000 csu302 303.9000 SYN30635 304.0000 PZE-105110067 306.0000 PZE-105110168 308.0000 umc1349 310.8000 myb3 319.9000 umc1221 329.5000 csu308 332.3000 umc1171 337.5000 umc1482 344.9000 bnl5.71a 348.1000 mmc0081 351.0000 phi333597 355.5000 umc1822 357.7000 umc2026 358.1000 mmp47 363.3000 umc1264 366.0000 umc1155 370.9000 nbp35 373.7000 mmp90 374.5000 mmp104 400.1000 umc126a 411.0000 mmc0481 417.2000 umc54 421.8000 umc1752 429.0000 umc1680 434.1000 umc1524 434.1000 php20531 434.7000 npi458a 440.1000 bnlg609 441.2000 rz567a 451.8000 php20566 452.5000 mmp169 456.8000 cdo507b 460.6000 rz273b 460.6000 phi085 469.2000 npi442 470.5000 umc108 477.1000 bnlg1118 531.0000 umc1072 539.8000 mmp118 540.9000 bnl5.24a 549.3000 bnlg118 549.3000 bnlg1597c 553.6000 mmp170 559.5000 umc1792 565.5000 npi288a 570.1000 php20523b 575.8000 umc1225 578.2000 mmp175 582.3000 umc104b 594.0000 rz446b 595.7000 php10017 600.4000 umc1153 607.7000

C6; 135 marker
umc49f 0.0000 umc1143 17.5000 bnlg161b 23.2000 bnlg238 23.2000 rz143a 46.2000 umc85a 52.4000 isu085a 53.6000 umc1606 55.2000 cdo1173c 57.1000 bnlg1371 58.7000 bnl6.29a 59.3000 bnlg1867 63.3000 umc1229 65.8000 php20528 70.4000 cdo545 71.7000 php20854 72.4000 uck1 75.5000 umc1133 78.0000 uaz232b 79.2000 mmp160 80.8000 mmp76 82.7000 ufg69 83.7000 mmp20 84.8000 mmp10 89.3000 mmp4 95.1000 mmp108b 97.2000 y1ssr 99.4000 umc1006 103.9000 mir1 106.0000 rz242a 106.7000 umc1656 112.3000 mmp117 118.4000 mmp51 122.1000 umc1257 124.6000 psr129b 126.8000 psb108 130.0000 umc1595 132.6000 csu923 137.6000 umc65a 155.9000 umc1796 163.5000 rz476d 165.3000 npi223a 170.3000 umc1105 173.0000 umc1979 174.3000 umc1857 177.2000 umc1014 185.5000 umc2006 202.9000 PZE-106063012 205.5000 PZE-106063281 206.0000 SYN15518 206.5000 PZE-106064381 207.0000 PZE-106065211 207.5000 PZE-106065285 208.0000 SYN25308 208.5000 SYN23913 209.0000 SYN23909 209.5000 SYN29430 210.0000 PZE-106066077 210.5000 SYN29431 211.0000 SYN34928 211.5000 isu061f 213.1000 PZE-106066563

214.5000 PHM1190.3 214.7000 SYN13424 214.7000 PZE-106067758 214.9000 PZE-106068040 214.9000 PZE-106067224 214.9000 PZE-106067891 215.0000 umc21 215.1000 SYN15753 216.1000 PZE-106068309 217.2000 PZE-106068510 218.3000 PZE-106069625 219.4000 SYN26935 220.5000 PZE-106069853 224.5000 PZE-106070038 225.5000 uaz280c 225.8000 SYN7542 226.5000 umc1250 228.6000 PZE-106070575 230.5000 PZE-106072681 231.5000 csu481 235.2000 umc1352 245.6000 umc1413 251.3000 umc2141 260.9000 umc1379 262.6000 npi560 264.7000 umc1388 267.5000 mmp62 269.5000 npi616a 273.4000 npi252 275.4000 psb107b 277.3000 bnlg1174 278.7000 bnlg1702 284.0000 phi078 286.2000 umc1020 288.8000 npi608 305.7000 uaz121a 325.6000 mmp145 330.6000 bnlg1732 337.0000 rz444d 339.0000 mmp150 341.6000 umc38a 349.0000 umc1912 351.9000 umc1859 354.6000 umc1463 357.1000 umc1762 357.3000 umc2162 361.7000 bcd738a 363.6000 lim379 378.9000 lim151 381.6000 psr162 386.4000 umc2170 392.9000 asg6a 399.4000 umc132a 401.8000 nfa102 405.5000 phi299852 408.3000 phi070 410.3000 umc1490 424.4000 npi419a 432.3000 mmp113 443.3000 umc2165 448.9000 bnlg1759a 449.4000 umc1350 450.5000 bnlg1740 456.3000 umc62 459.1000 npi561 470.2000 bnlg1136 475.9000 php20599 476.9000 mmp105 478.7000 csu897 480.5000 agp2 480.5000 umc2059 486.8000 cdo345c 489.8000 cdo202a 492.7000

C7; 116 marker
umc2177 0.0000 csu582 2.7000 umc1241 13.8000 umc1378 27.4000 umc1672 43.8000 umc1694 45.0000 umc1426 47.8000 bnlg2132 53.3000 asg8 61.4000 php20581a 71.7000 umc1159 85.0000 mmp18 106.4000 mmp81 108.8000 umc2160 111.5000 umc1066 115.4000 umc1270 116.5000 umc1577 118.2000 asg34a 122.4000 gta101a 139.0000 umc1401 142.4000 umc1016 143.6000 umc1978 145.0000 npi600 149.8000 crt2 153.0000 phi034 158.0000 bnlg1094 158.6000 psr371b 160.7000 uaz187 161.8000 rz698d 163.7000 bnlg1247 164.4000 bnlg2233 164.6000 bnlg1380 166.2000 bnlg2203 168.7000 lim333 172.1000 umc1932 181.0000 umc1983 197.9000 umc2142 199.9000 umc1138 201.3000 umc1929 202.7000 umc1787 206.0000 umc2092 206.5000 umc1393 212.0000 umc5b 215.1000 isu086 216.1000 ufg54 234.1000 cdo412b 239.0000 bnlg1808 239.9000 umc116a 242.4000 mmp127 243.7000 mmc0411 246.2000 umc1713 251.9000 umc1015 253.5000 bcd926a 260.9000 bnl15.21 263.3000 mmp177c 268.7000 umc1450 270.3000 umc1987 272.3000 mmp46 273.9000 bnlg1070 277.0000 bnlg434 277.6000 npi394 284.9000 mmp152 298.9000 umc1660 303.9000 npi389 308.3000 umc56 315.8000 umc110a 318.7000 umc1837 322.8000 rz404 327.9000 bnlg155 330.8000 umc111b 332.9000 umc1134 335.1000 psr371a 335.7000 ndk1 336.5000 bnlg2271 337.7000 umc1112 339.0000 umc1324 341.3000 umc1888 344.2000 bnlg1805 345.7000 isu150 346.7000 tif1 359.6000 psr135a 363.7000 umc1936 365.8000 umc1301 365.8000 umc254 368.4000 umc1710 370.8000 umc1251 372.4000 asg32 376.8000 ufg17 387.8000 bnlg1666 390.8000 npi240a 392.6000 npi263 393.5000 npi352 400.1000 bnl8.29c 402.5000 umc1029 404.9000 bcd349 424.7000 umc1708 431.5000 csu8 435.5000 umc1768 440.8000 bnlg2259 448.9000 umc1295 454.5000 ufg57 457.3000 umc1412 478.6000 umc245 491.7000 npi380 493.4000 npi433 496.4000 php20909b 498.4000 phi069 504.9000 cdo938d 541.2000 umc1406 549.9000 umc35a 551.2000 umc1407 551.2000 ufg39 553.9000 umc168 559.2000 phi116 562.6000 php20020 563.1000 npi611a 566.0000

C8; 107 marker
npi220a 0.0000 csu319 5.9000 rz382b 6.7000 npi114a 10.1000 umc1139 26.8000 umc2042 30.3000 mmp148 31.8000 umc1592 33.8000 bnl13.05a 44.3000 umc1414 48.0000 umc1327 55.2000 umc1483 85.9000 mmp85 101.8000 bnlg1194 107.3000 npi110a 112.4000 cdo460 115.7000 mmp57 117.0000 umc1304 129.2000 bnlg2235 133.0000 bcd1823a 136.2000 mmp166 139.6000 npi585a 149.1000 umc1974 153.4000 psr598 156.7000 cdo328 159.3000 umc1913 160.9000 csu329 176.0000 umc124a 176.7000 umc1530 179.6000 mmp120 191.1000 mmp72

194.2000 mmp158b 198.0000 umc32b 199.2000 bnlg2082 200.7000 rz244a 201.6000 bnlg1834 202.5000 umc1807 203.5000 umc1157 203.7000 umc1904 204.3000 npi260b 208.7000 cdo1160a 213.3000 umc1910 214.6000 mmp195f 218.3000 cdo202e 222.5000 php3818 223.8000 umc1415 226.3000 umc1470 228.9000 umc1984 232.6000 phi100175 240.1000 umc1735 245.3000 umc1457 250.0000 phi121 255.2000 php20714 256.7000 umc2154 260.7000 umc1460 269.6000 umc1858 275.5000 bnlg2046 278.3000 gta101d 286.9000 bnl2.369 292.5000 umc1130 293.2000 umc1309 300.2000 mmp15 305.6000 umc1959 309.7000 umc1562 310.9000 umc1263 311.5000 psb107a 314.1000 ufg80 317.0000 ufg74 320.9000 bnlg666 324.5000 umc89a 327.0000 umc12a 329.9000 bnlg1651 331.6000 umc1889 332.1000 umc1340 334.9000 hda103 336.4000 umc1316 338.9000 isu114 339.9000 bnl12.30a 342.8000 umc1149 371.2000 umc1728 389.7000 umc1905 396.9000 bnlg1031 412.4000 npi268a 416.5000 bnlg1065 418.1000 rz538a 421.3000 umc1607 423.8000 bnlg1823 440.7000 psy2 447.0000 umc1268 452.0000 lim301 461.6000 bnlg1828 464.1000 npi414a 467.1000 mmp64 472.2000 php20793 479.2000 umc1005 483.0000 umc1933 496.3000 mmp146 500.7000 umc1673 502.9000 npi107 512.2000 npi112b 514.2000 phi015 517.4000 csu146b 521.2000 agrr21 525.9000 phi233376 548.1000 umc1638 560.9000 umc1916 566.0000 bnlg1131 567.5000

C9; 125 marker
umc1957 0.0000 umc109 5.0000 bnlg1724 11.8000 npi253a 14.0000 umc1370 17.7000 bnlg2122 21.3000 umc1867 24.3000 php10005 28.8000 lim343 46.2000 ufg41 50.4000 bnlg1810 62.2000 bnlg1583 62.2000 c1 64.5000 umc1809 65.0000 sh1 79.1000 umc1588 80.9000 umc1967 82.9000 umc1596 85.4000 bz1 88.7000 umc1170 97.1000 csu471 101.9000 isu111b 112.6000 umc1636 124.5000 mmp162 132.6000 bnlg244 136.2000 bnlg1401 141.1000 mmp77 146.6000 mmp30 156.0000 umc1698 163.8000 lim286 176.3000 phi022 177.8000 umc1634 179.3000 umc1258 181.8000 umc1586 185.8000 lim101 188.4000 ufg71 190.5000 mmp170b 194.6000 psr160d 202.0000 psr160c 205.1000 rz273c 208.3000 umc81 210.7000 rz953 210.7000 bcd1421 211.7000 php20052 212.6000 bnl5.10 213.3000 csu623 214.8000 umc1191 217.0000 mmp2 219.8000 asg63a 222.5000 umc1271 224.4000 umc1691 228.0000 umc1688 231.5000 umc20 233.1000 umc1700 235.7000 umc1743 237.9000 umc114 238.2000 bnl5.04 239.9000 rz682 242.0000 lim99b 243.6000 bnl7.13 248.7000 umc1570 249.8000 umc1571 251.0000 lim166 252.2000 bnlg1209 257.0000 psr547 262.7000 psr129a 266.9000 umc1107 269.6000 bnlg1159b 270.8000 gta101c 273.9000 bnlg1012 281.8000 ufg70 284.0000 npi580a 286.1000 isu041a 288.7000 ufg68 290.0000 umc1492 291.8000 umc1120 293.7000 phi032 295.3000 mmp96 296.3000 mmp37 298.2000 umc2121 299.6000 umc38c 300.9000 umc95 304.5000 umc140b 305.8000 umc1078 306.5000 lim458 308.1000 ufg13a 309.9000 ufg64 313.2000 ufg63 315.1000 php20554 322.0000 umc1231 325.9000 mmp41 327.6000 mmp151d 332.7000 ufg67 345.2000 ufg47 346.0000 umc2095 354.9000 csu634 358.1000 rz574b 359.2000 npi427a 362.1000 ufg24 367.6000 umc2134 379.4000 npi443 385.0000 mmp142 396.7000 npi439b 406.8000 mmp132 408.6000 asg44 416.3000 mmp131 433.6000 csu93a 436.9000 ufg75c 450.2000 mmp168 459.5000 umc1366 462.6000 bnl5.09a 469.0000 mmp110 473.3000 bnl14.28a 477.4000 isu049 479.0000 umc1789 482.3000 asg12 484.2000 umc1675 488.5000 bnlg619 501.7000 mmp136 503.7000 umc2089 510.0000 umc2131 514.1000 psb596 514.9000 mmp171a 524.3000 umc1137 551.2000 umc1505 581.6000

C10; 103 marker
mmp48a 0.0000 mmp48b 11.0000 umc1380 16.7000 php20626 19.3000 psr119c 23.6000 bnl3.04 24.9000 php20753a 25.3000 phi041 26.2000 php20075a 30.1000 csu1061b 47.9000 umc2053 62.3000 umc2018 67.2000 umc1152 77.5000 umc1576 84.0000 umc2034 94.0000 phi059 104.4000 isu085b 105.6000 umc2069 116.6000 umc130 120.7000 csu625 121.1000 npi105a 123.9000 umc18b 124.8000 gcsh1 128.9000 lim2 134.2000 bcd1072b 138.2000 php06005 139.3000 umc1962 141.4000 bnlg210 144.1000 umc1367 144.5000 uaz116 145.4000 php20646

145.8000 umc1381 147.8000 umc2016 151.6000 bcd147 152.6000 psb527c 154.1000 umc1345 156.0000 ufg59 158.7000 umc1179 164.1000 umc1239 168.9000 psr690 171.4000 npi445a 172.8000 bnlg1712 173.4000 umc155 176.9000 phi050 177.4000 umc1336 178.7000 fgp1 184.9000 umc64a 192.9000 umc1995 196.5000 umc1246 198.8000 mmp16 202.2000 umc1077 204.0000 umc1090 204.2000 phi062 206.0000 psb527d 209.5000 umc2163 215.1000 umc1053 216.6000 umc1911 218.0000 asg2 221.6000 umc1330 230.6000 php15013 234.2000 umc1115 238.7000 umc1272 242.2000 umc1930 249.7000 php20719a 251.2000 umc259a 251.8000 npi578 252.0000 umc1677 254.1000 isu058b 257.9000 mmp12 270.0000 bnlg1074 274.8000 bnlg1250 278.2000 ufg81 283.8000 csu745a 286.1000 umc1506 287.5000 ufg37 295.1000 ufg28a 295.1000 umc1477 308.9000 bnl10.13a 318.9000 bnlg1028 323.1000 psb365a 326.0000 bnl17.02 330.5000 umc2122 335.1000 umc1993 353.2000 bnlg2190 354.9000 ufg62 359.2000 bnl7.49a 380.2000 agrr37c 384.8000 umc1196 387.4000 umc1084 388.2000 bnlg1677 391.5000 npi208b 393.8000 mmp181 403.6000 bnlg1839 405.3000 umc1176 407.3000 bnlg1360 408.6000 npi254b 409.8000 bnlg1450 422.6000 php20568a 435.5000 umc2021 444.4000 umc2126 452.1000 isu053b 461.5000 asg19b 463.0000 csu48 472.1000
